# Quantitatively mapping local quality of super-resolution microscopy by rolling Fourier ring correlation

**DOI:** 10.1038/s41377-023-01321-0

**Published:** 2023-12-14

**Authors:** Weisong Zhao, Xiaoshuai Huang, Jianyu Yang, Liying Qu, Guohua Qiu, Yue Zhao, Xinwei Wang, Deer Su, Xumin Ding, Heng Mao, Yaming Jiu, Ying Hu, Jiubin Tan, Shiqun Zhao, Leiting Pan, Liangyi Chen, Haoyu Li

**Affiliations:** 1 Innovation Photonics and Imaging Center, School of Instrumentation Science and Engineering, Harbin Institute of Technology, Harbin, China; 2 Key Laboratory of Ultra-Precision Intelligent Instrumentation of Ministry of Industry and Information Technology, Harbin Institute of Technology, Harbin, China; 3 Biomedical Engineering Department, International Cancer Institute, Peking University Cancer Hospital and Institute, Health Science Center, Peking University, Beijing, China; 4 The Key Laboratory of Weak-Light Nonlinear Photonics of Education Ministry, School of Physics and TEDA Institute of Applied Physics, Frontiers Science Center for Cell Responses, Nankai University, Tianjin, China; 5 State Key Laboratory of Membrane Biology, Beijing Key Laboratory of Cardiometabolic Molecular Medicine, Institute of Molecular Medicine, National Biomedical Imaging Center, School of Future Technology, Peking University, Beijing, China; 6 Department of Control Science and Engineering, Harbin Institute of Technology, Harbin, China; 7 School of Mathematical Sciences, Peking University, Beijing, China; 8 Unit of Cell Biology and Imaging Study of Pathogen Host Interaction, The Center for Microbes, Development and Health, Key Laboratory of Molecular Virology and Immunology, Shanghai Institute of Immunity and Infection, Chinese Academy of Sciences, Shanghai, China; 9 School of Life Science and Technology, Harbin Institute of Technology, Harbin, China; 10 PKU-IDG/McGovern Institute for Brain Research, Beijing, China; 11 Beijing Academy of Artificial Intelligence, Beijing, China; 12 Frontiers Science Center for Matter Behave in Space Environment, Harbin Institute of Technology, Harbin, China; 13 Key Laboratory of Micro-Systems and Micro-Structures Manufacturing of Ministry of Education, Harbin Institute of Technology, Harbin, China

**Keywords:** Super-resolution microscopy, Microscopy, Optical physics

## Abstract

In fluorescence microscopy, computational algorithms have been developed to suppress noise, enhance contrast, and even enable super-resolution (SR). However, the local quality of the images may vary on multiple scales, and these differences can lead to misconceptions. Current mapping methods fail to finely estimate the local quality, challenging to associate the SR scale content. Here, we develop a rolling Fourier ring correlation (rFRC) method to evaluate the reconstruction uncertainties down to SR scale. To visually pinpoint regions with low reliability, a filtered rFRC is combined with a modified resolution-scaled error map (RSM), offering a comprehensive and concise map for further examination. We demonstrate their performances on various SR imaging modalities, and the resulting quantitative maps enable better SR images integrated from different reconstructions. Overall, we expect that our framework can become a routinely used tool for biologists in assessing their image datasets in general and inspire further advances in the rapidly developing field of computational imaging.

## Abstract (web summary)

We develop a rolling Fourier ring correlation (rFRC) method to evaluate the reconstruction uncertainty at super-resolution scale, detecting the spatial resolution heterogeneity of super-resolution microscopy along different dimensions.

## Graphical abstract

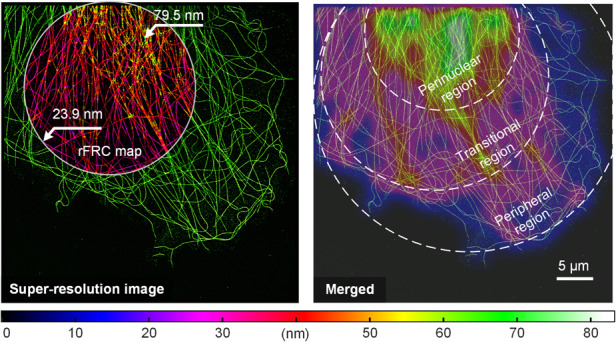

## Introduction

By implementing fluorescent probes and combining specific excitation and emission protocols, super-resolution (SR) fluorescence microscopy breaks the diffraction limit of resolution (200–300 nm)^1–3^, in which many methods heavily depend on image calculation and processing that retrieve the SR information^1,4^. Intrinsically, the noise and distortions in raw images caused by the photophysics of fluorophores^5–7^, the chemical environment of the sample^5,6,8^, and the optical setup conditions^6,9–12^, may influence the qualities of the final SR images^13–15^. Because these factors are related to specific experimental configurations, a reliable and reference-free estimation of the image quality is invaluable to subsequent analysis, especially at the SR scale.

Although the artifacts can be assessed by imaging standard reference structures^16,17^ or benchmarking against other higher resolution imaging methods^18,19^, people have developed various ingenious methods aiming to evaluate the SR image qualities without referencing ground-truth to the best of their abilities. For example, the HAWKMAN (Haar wavelet kernel analysis method for the assessment of nanoscopy)^20^ was developed for the assessment of single-molecule localization microscopy (SMLM)^18,21,22^, and the SIM-check^23^ was provided for specially quantifying the structured illumination microscopy (SIM) images. In addition to these domain-specific methods, the Fourier ring correlation (FRC)^24^ was developed to evaluate the global effective resolution in general, describing the highest reliable cut-off frequency of an image. This effective resolution, or equivalently the spectral signal-to-noise ratio (SNR), is one crucial SR image quality metric, reflecting the authentic resolvability or the uncertainty^25^. However, an unavoidable issue is the heterogeneity of resolution, and in other words the local resolution may vary dramatically over the imaging field. For example, in SMLM systems^18,21,26^, the practical resolutions at different local regions are generally determined by the corresponding molecule active intensity and density, as well as the local background level^13^. To measure this resolution heterogeneity, the block-wise FRC calculation^24,27^ was introduced, but it is still too coarse to describe the SR scale spatial separation of the resolution variation. Therefore, the upscaled resolvability of SR imaging requires a more elaborate evaluation.

Here, we propose a rolling Fourier ring correlation (rFRC) method to draw the resolution heterogeneity directly in the SR domain, which allows for mapping at an unprecedented SR scale and seamlessly correlates the resolution map with the SR content. The variations of different SR reconstruction methods are usually on a fine scale, and our rFRC provides a prerequisite for assessing these methods objectively. Thus, it enables advancing process procedures to improve image restoration quality, such as fusing SMLM images reconstructed by different algorithms to yield SR images with better quality. Although we are limited to calculate the errors as without ground-truth comparing, we can measure the uncertainties by this rFRC to uncover the errors contained in the corresponding SR images. In other words, the lower spectral SNR (effective resolution) gives a higher probability of the error existence^25^, and thus we can use it to represent the uncertainty revealing the error distribution (Supplementary Note 1).

As a model-independent assessment, the rFRC using two independent captures may fail to identify regions that were always incorrectly restored during different reconstructions, possibly due to systematic image processing bias (model bias). On the other hand, the resolution-scaled error map (RSM)^27^ can evaluate reconstruction errors against the simultaneously acquired high SNR wide-field reference, assuming a spatially invariant Gaussian kernel and homogenous illumination. However, RSM suffers from false-negative identifications when the assumptions fail, and its detectable error scale is limited by the diffraction barrier. In this sense, RSM can only estimate the large-scale errors, such as the complete absence and distortion of structures, possibly induced by model bias, which can be a complementary module. In this work, we also accompany our rFRC with a truncated RSM, namely PANEL (Pixel-level ANalysis of Error Locations), pinpointing the regions with low reliability for subsequent biological profiling. Then we applied our quantitative maps in many imaging approaches, including SMLM, SR radial fluctuations (SRRF)^28^, SIM^15^, and deconvolution^29,30^, demonstrating its effectiveness and stability. To expect our method can be a routinely used local quality evaluation tool, it has been implemented as an open-source framework; the related MATLAB and Python libraries and the out-of-the-box Fiji/ImageJ^31^ plugin are available on GitHub (Methods).

## Results

### rFRC mapping and PANEL pinpointing

Systematically, the reconstruction qualities of the corresponding SR modalities are influenced by two types of degradation, i.e., the model bias and the data uncertainty^32^. The model-bias-induced errors (model error) are primarily caused by the difference between the artificially created estimation model and its physical, real-world counterpart, which can be detected and minimized by carefully calibrating the optical microscopy system and measuring its characteristics^12,33,34^. On the other hand, the data uncertainties are mostly introduced by joint effects of the noise condition and sampling capability of the hardware equipment, such as the sensors and cameras in microscopy systems. In other words, the reconstructions from the inevitably deviated observations (left panel of Supplementary Fig. S13a) may also break away from the real-world objects in the target SR domain (right panel of Supplementary Fig. S13a). Therefore, the data uncertainties are generally inevitable, hard to be suppressed by system calibrations, and free from the model, which may be more critical in quantitative biological-image analysis.

Heuristically, we can capture statistically independent images of the object by imaging the identical object with the same configurations. The data uncertainties can be highlighted by the difference between these individual reconstructions (Supplementary Fig. S13b, Supplementary Note 1). In this sense, the conventional pixel-wise spatial evaluation methods, such as spatial subtraction or standard deviation of the individually reconstructed results may quantify the distance between the recorded images. However, by measuring the ‘absolute differences’, the subtraction between this image pair is susceptible to the intensity fluctuation and subpixel structural motions^35^, producing false negatives in distance maps that overshadow genuine errors. To minimize the false negatives, we repurposed the Fourier ring correlation (FRC)^24^ to quantify the distance between two individual SR reconstructions in the Fourier domain. By calculating the ‘relative error’ or ‘saliency-based error’, the FRC is insensitive to intensity fluctuations or structure movements, thus more robust (Supplementary Note 1, Supplementary Fig. S14).

**Fig. 1:**
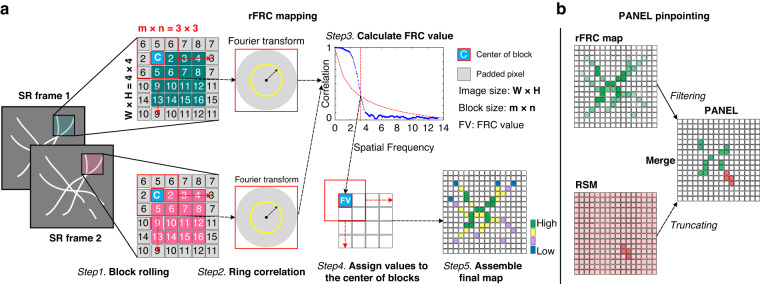
Overview of rFRC mapping and PANEL pinpointing. **a** The workflow of the rFRC map. Step 1, the symmetrically padded (gray pixels) two input images are clipped to small subsets for FRC calculation. The center pixel with an intensity lower than the background threshold will be skipped; otherwise, the following Steps 2–5 will be executed. Steps 2–3, FRC calculation, ring correlation in Fourier domain (Step 2), and FRC resolution determination (Step 3). Step 4, assign the obtained FRC resolutions to the corresponding center pixels. Step 5, assemble the final rFRC map and render it with the corresponding color map. **b** PANEL pinpointing. To highlight regions with low reliability, the rFRC map with values under the Otsu-determined threshold; and the normalized RSM (see also Supplementary Fig. S4) with values under 0.5 will be filtered. Its abstract version can be seen in Supplementary Fig. S1

To provide local distance measurements, we transformed the conventional FRC framework into a rolling FRC (rFRC) map, which directly evaluated spatial information at the SR scale (Fig. 1a, Methods). The rFRC calculation is similar to that of a moving filter on an image. We assigned the corresponding FRC resolution in each block by sliding a window through the image. (i) To calculate the FRC of pixels at the image boundaries, we padded the input image symmetrically around a half size of the block (Step 1, Fig. 1a). (ii) To eliminate background-induced false negatives, we avoided calculating areas indistinguishable from the background (Methods, Supplementary Fig. S2), in which we assigned the calculated FRC resolution to the center pixel of each block only if its mean intensity was larger than a given threshold (Steps 2–4, Fig. 1a). To avoid overconfident and unstable determinations from small image blocks, in this work we used the 3σ curve^36^ as criterion (Methods, Supplementary Note 2.1). (iii) Afterward, the same procedure was repeated block by block throughout the entire image. Compared to the block-wise FRC map^27^, our rFRC achieves the pixel-level mapping sensitivity by rolling operation; eliminates the potential false-negative by background threshold; and increases the robustness of small image estimation by using 3σ curve as criterion. Using the above rFRC as the metric we can quantitatively map the uncertainties in the SR reconstructions at their SR scale (Supplementary Note 2.2). Notably, this SR scale representation of the local qualities essentially denotes the measurement sensitivity (the direct evaluation in SR domain) rather than the SR imaging resolution (Rayleigh resolution) (Supplementary Note 2.2).

In addition to local quality assessment, we calculate two global metrics, the rFRC value, a dimensionless metric with values starting at 0 reflecting the deterioration rate across the imaging field, and the rFRC resolution, representing the averaged resolution (Methods). We also offer two optional colormaps that may be more suitable for human intuition^37^ to display the uncertainties (shifted Jet, sJet; and black Jet, bJet) (Step 5, Fig. 1a, Methods, Supplementary Fig. S3a). Beyond that, we realized that the rFRC may not identify the regions that were always incorrectly restored during different reconstructions due to the model bias. For example, if the two reconstructed images lost an identical component, the rFRC may indicate a false positive in the corresponding region. To moderate this issue, we combined a modified RSM (Methods, Supplementary Fig. S4) with our rFRC to constitute the PANEL (Fig. 1b, Methods), for pinpointing such regions with low reliability. As small intensity fluctuations can lead to potential false negatives, we truncated the RSM with a hard threshold (0.5, Methods), only including prominent artifacts such as misrepresentations or the disappearance of structures. To filter the regions with high quality (high FRC resolution), we adopted the Otsu-based^38^ segmentation to highlight regions giving a higher probability of the error existence (Methods, Supplementary Fig. S3b). We then merged the filtered rFRC map (green channel) and RSM (red channel) to create the composite PANEL map (Fig. 1b). Note that our PANEL cannot fully pinpoint the unreliable regions induced by the model bias at present, which would require more extensive characterization and correction routines based on the underlying theory of the corresponding models^12,34,39,40^.

### Validating with SMLM simulations

**Fig. 2:**
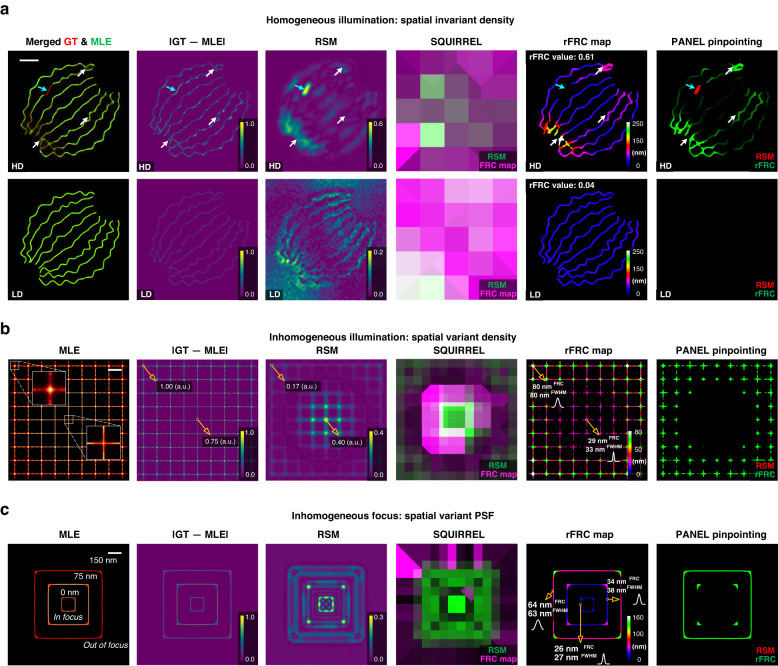
Systematic validations using SMLM simulations. **a** Simulations of 2D-SMLM with homogeneous illumination (inducing overall spatial invariant active density), with high-density (HD, top) and low-density (LD, bottom) emitting fluorophores in each frame. Cyan and white arrows represent the errors found by the RSM or rFRC map, respectively. **b** 2D-SMLM simulation with inhomogeneous illumination (high intensity in the center and decreasing illumination toward the edges). **c** 2D-SMLM simulation with inhomogeneous focus (in focus at the center and defocusing toward the edges). From left to right: Merged MLE reconstructions (green channel) and ground-truth images (red channel); Spatial subtractions between ground-truth images and MLE reconstructions; Spatial subtractions between wide-field ground-truth images and wide-field images generated from the MLE reconstructions, a.k.a. the RSM; The full SQUIRREL map (RSM corresponding to the green channel and FRC map to the magenta channel); The rFRC maps of two MLE reconstructions from odd frames and even frames, respectively; The full PANEL visualizations (RSM corresponding to the red channel and rFRC map to the green channel). Scale bars: (**a**) 500 nm; (**b**, **c**) 1 μm

To test our quantitative maps with known ground truth, we used simulated datasets of SMLM from the EPFL challenge^13^ (Methods, Fig. 2a). These datasets consisted of high-density (HD, 361 frames) and low-density (LD, 12,000 frames) emitters per frame to simulate excessively low or optimal illumination intensity conditions. The images were divided into two statistically independent subsets (odd and even frames), yielding two SR reconstructions obtained using the maximum likelihood estimation (MLE)^41^, for our rFRC mapping (Methods, 5th column in Fig. 2a). Between MLE reconstructions and ground-truth images, their differences in space indicate the locations and scales of different artifacts (first two columns in Fig. 2a). From this spatial difference and localization uncertainty maps (Supplementary Fig. S5a, S5b), we found that the reconstructed SR image under the HD condition was much more blurred than that of the LD condition, possibly due to more overlapping emitters being excited simultaneously. This was affirmed by the larger rFRC value (Methods) of the HD-MLE image than that of the LD-MLE image (0.61 versus 0.04, 5th column in Fig. 2a), in which the rFRC map uncovered all the subtle errors (as pointed by white arrows). In contrast, distinctly, the previous RSM and full SQUIRREL^27^ map (3rd and 4th columns in Fig. 2a) cannot detect such subtle errors, and RSM is influenced by noise-induced random intensity fluctuations. On the other hand, we noted that rFRC failed to detect the missing part in filaments, mimicking defective local illumination or labeling (cyan arrows, Fig. 2a). That was revealed by the truncated RSM, highlighting the necessity of PANEL combination for pinpointing different types of errors (last column in Fig. 2a).

To demonstrate the dependence of the SMLM reconstruction quality on the illumination intensity, we synthesized a regular grid illuminated by a Gaussian beam with high power in the center and low power toward the edges (Methods, Supplementary Fig. S5c, S5d). Under this circumstance, molecule blinkings at the center were better separated temporally than those at the edges^27^ (1st column in Fig. 2b, Supplementary Fig. S5d), which was clearly revealed on the rFRC map (5th column in Fig. 2b). Interestingly, examining the different filaments both at center (33 nm FWHM versus 29 nm FRC resolution) and edge (80 nm FWHM versus 80 nm FRC resolution) regions, we found these measured FWHMs (full width at half maximum) nicely match the corresponding estimated resolutions. In contrast, because the space-invariant reconstructed PSF assumption did not hold up here, RSM and full SQUIRREL map incorrectly provided the estimated errors (3rd and 4th columns in Fig. 2b, 0.40 a.u. at the center and 0.17 a.u. at the edge, Supplementary Fig. S5e), which was opposite to the reference (2nd column in Fig. 2b, 0.75 a.u. at the center and 1.00 a.u. at the edge).

Next, we extended our test to the issue of spatially variant PSF (Fig. 2c), which is induced by gradually defocusing in three stages toward the edges. It is conceivable that the localization precision will drop correspondingly due to this defocusing effect. Because of the spatially variant PSF, the RSM and full SQUIRREL map (3rd and 4th columns in Fig. 2c) incorrectly provided the opposite evaluation and visualization for reconstruction quality. We demonstrated that our rFRC map and full PANEL map accurately represented this spatially variant resolution across the imaging field, and the estimated FRC resolutions closely fit the three-stage distribution that manually measured from the FWHM values, i.e., 26 nm, 34 nm, and 64 nm in FRC values versus 27 nm, 38 nm, and 63 nm in FWHM values.

Although the RSM is incompatible with volumetric datasets, the rFRC can be directly extended to a 3D version when applying plane-by-plane calculations (Methods). Here, we also presented the simulated 3D dataset from the EPFL SMLM challenge^42^, including both LD and HD emitters (Methods, Supplementary Fig. S6). Similarly, compared to the reconstruction with LD emitters per frame, the rFRC analysis demonstrated lower quality with HD emitters (3D rFRC value *LD*: 4.9, *HD*: 7.0, Supplementary Fig. S6b), confirming the real experimental experience.

### Detecting resolution heterogeneity along different dimensions

**Fig. 3:**
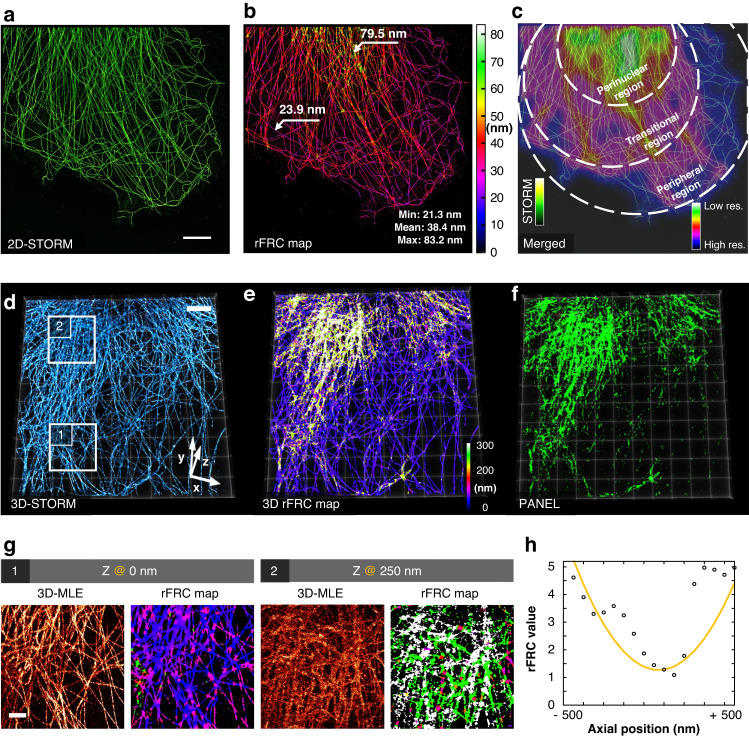
Super-resolution images from localization microscopies evaluated by rFRC. **a** STORM result of α-tubulin labeled with Alexa Fluor 647 in a COS-7 cell (left). **b** rFRC map of (**a**). **c** The merged view of STORM result (green hot) and Gaussian averaged rFRC map (shifted jet) for highlighting low-quality regions. **d** 3D-STORM results (COS-7 cells, α-tubulin labeled with Alexa Fluor 647). **e** 3D rFRC map of (**d**). **f** PANEL after the Otsu threshold of (**e**). **g** Corresponding magnified horizontal sections of the 3D-STORM (left) and rFRC (right) volume of the white boxes in (**d**). **h** The curve of the rFRC values along with the axial positions. Scale bars: (**a**, **d**) 5 μm; (**g**) 2 μm

Next, we examined the experimental SMLM microtubule datasets (Methods, Fig. 3, Supplementary Fig. S7). As visualized by the rFRC, the SR microtubule images obtained by large-field STORM^43^ (Fig. 3a), small-field SMLM^13^ (Supplementary Fig. S7a, S7b), and SRRF^28^ (Supplementary Fig. S7c) demonstrated significantly lower resolutions at filament intersections (Fig. 3b, Supplementary Fig. S7) and perinuclear region of the cell (Fig. 3c). This is because the regions with more complex structures will exhibit more simultaneous emitters per area, inducing a relatively degraded resolution. In addition to this spatially variant density, the potential out-of-focus effects in the deeper perinuclear region will also decrease the localization precision. In detail, as can be seen in Fig. 3c, the perinuclear region contains the most dense cytoskeleton, which shapes the thickest space, and its surrounding region is the transitional subregion with the microtubules becoming sparser. The peripheral subregion is further out, which exists in the thin perimeter areas of the cell^44^, and the microtubules appear as an expansive network. Interestingly, such three-stage structural distribution-induced resolution heterogeneity is successfully mapped by our rFRC (Fig. 3c).

We further provided the rFRC map procedure for the 3D-STORM^43^ reconstruction (Fig. 3d), in which significant uncertainties also occurred at the perinuclear region (Fig. 3e, f). In addition to the laterally varied qualities, it is found that the resolution also fluctuates along the axial dimension. Under this 3D configuration, we found that the overall most accurate axial planes for microtubule (Fig. 3g, h) and actin filaments (Supplementary Fig. S8) were located at the focal planes. Notably, we directly applied rFRC mapping on volumetric datasets in a slice-by-slice manner to visualize the quality variations on each plane. The 3D extension of our method requires 3D rolling operation and the Fourier shell calculation to further incorporate axial information and measure the anisotropy of lateral and axial resolutions^45^.

### Optimal fusion of SMLM

**Fig. 4:**
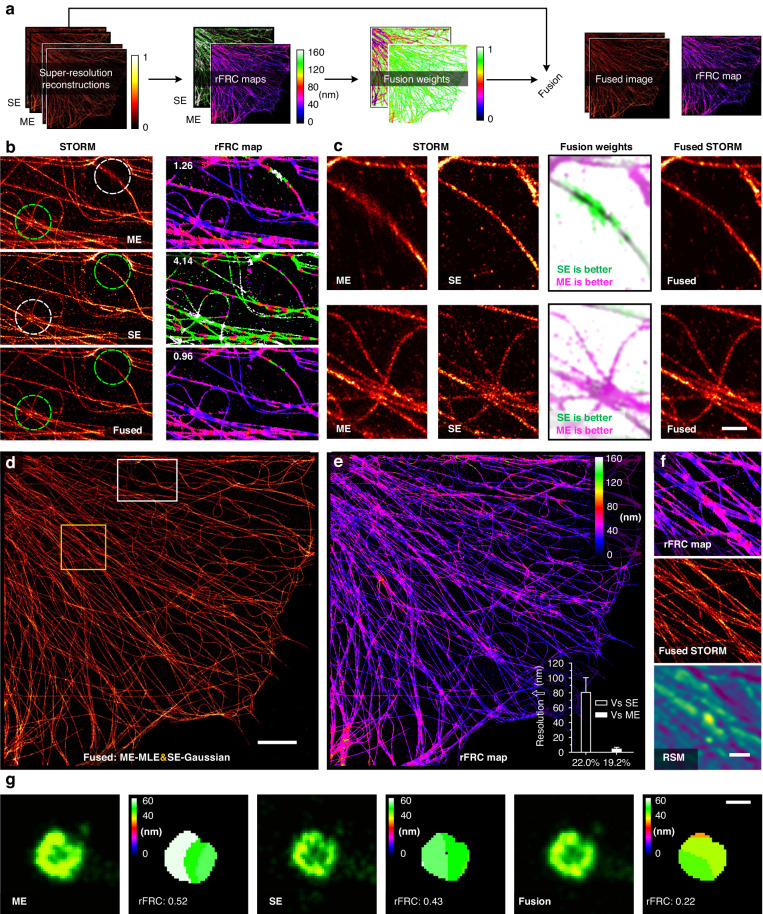
STORM fusion using the rFRC map. **a** Schematic of the STORM fusion. ME: Multi-emitter MLE result; SE: single-emitter Gaussian fitting result. **b** STORM results (COS-7 cells, α-tubulin labeled with Alexa Fluor 647, left) and their rFRC maps (right) are shown from top to bottom, which are magnified views of the white box in (**d**). From top to bottom: ME result; SE result; the fused result from the ME and SE reconstructions. The corresponding rFRC values are marked on the top left of the rFRC maps. **c** Magnified views of the dashed circles in (**b**). From left to right: ME results, SE results, fusion weights (inverted rFRC maps of ME results and SE results merged as green and magenta channels, respectively), and fused STORM results. **d** The entire view of the fused STORM result (COS-7 cells, α-tubulin labeled with Alexa Fluor 647). **e** rFRC map of (**d**). The inset shows the improved resolution achieved by fusion compared with the SE (80.55 ± 1.52 nm at 22.0% region, hollow) and ME (4.28 ± 0.14 nm at 19.2% region, white solid) results. **f** Enlarged regions enclosed by the yellow box in (**d**). The results of the rFRC map, fused STORM, and RSM are shown from top to bottom. **g** Another representative example for SMLM fusion (COS-7 cells, heavy chain clathrin-coated pits labeled with Alexa Fluor 647, see also Supplementary Fig. S9). The results of ME, SE, and fusion are shown on the left, and the corresponding rFRC maps are demonstrated on the right. Error bars, s.e.m.; scale bars: (**b,** **c**) 500 nm; (**d**) 5 μm; (**f**) 1 μm; (**g**) 100 nm

Since all current reconstruction algorithms assume homogenous HD or LD emitters per frame, the heterogeneity of resolution is becoming a major problem^27^. By identifying positions of high localization uncertainty with rFRC map, we can compare the local performances of different restoration algorithms, and fuse different regional reconstructions (Methods, Fig. 4a). To do so, the resolution heterogeneity and potential artifacts can be minimized. By integrating a high-density simulated dataset reconstructed by the multi-emitter MLE (ME-MLE)^41^ and the FALCON (fast localization algorithm based on a continuous-space formulation)^46^, the fused image demonstrated better PSNR (peak signal to noise ratio), SSIM (structural similarity), and the rFRC values (Supplementary Fig. S5f-S5i). To further evaluate its performance in real samples, we analyzed immunolabeled α-tubulin filaments in fixed COS-7 cells imaged with 2D-STORM and restored them with either the ME-MLE or single-emitter Gaussian fitting^43^ (SE-Gaussian) (Methods, Fig. 4b). Although the ME-MLE method performed better at approximating complex structures (HD emitters) and provided a lower overall rFRC value, the SE-Gaussian algorithm seemed to excel in reconstructing some simple structures (LD emitters) (Fig. 4b, c). By combining regions with the lowest local rFRC values between reconstructions from either of the two algorithms (Methods, third columns in Fig. 4c), the new composite SR image demonstrated better visual quality and the lowest overall rFRC value (0.96 versus 1.26 and 4.14, Fig. 4b). Moreover, the fused image exhibited a more homogenous distribution of spatial resolution than that obtained either by ME-MLE or SE-Gaussian alone (Fig. 4d, e), reinforcing its superior performance in the entire FOV. Specifically, this rFRC map-guided image fusion led to a substantially improved resolution (the inset in Fig. 4e) in replaced regions than the SE-Gaussian method (80.55 ± 1.52 nm, hollow), and significant increases in local resolutions than the ME-MLE method (4.28 ± 0.14 nm, white solid). In contrast, because the RSM method was incapable of revealing errors of SR ranges, we found it failed to identify such intricate structures from the STORM image (Fig. 4f). Similarly, the rFRC was used to composite fusion to clathrin-coated pits (CCPs) in COS-7 cells under 2D-STORM (Fig. 4g). The merged SR image showed better quality and higher mean resolution (Fig. 4g, Supplementary Fig. S9).

### Evaluating diverse optical super-resolution microscopies

After establishing the validity and superiority of our method in SMLM, we extended our analysis to other non-pointillism SR methods. In theory, because the weight of the optical transfer function (OTF) decreases gradually with its spatial frequency, the noise will dominate the high-frequency components while the low-frequency deep inside the OTF support remains stable. The subsequent reconstructions will apply more amplifications at higher frequencies than lower ones, leading to significant fluctuations in high-frequency components, which renders intricate structures more profoundly affected by noise. Moreover, the variations of different SR reconstruction methods are usually on their SR scale, and thus an evaluation on the corresponding level is essential. Here, our rFRC offers a well-timed solution to detect these uncertainties at high spatial frequencies. In the following applications, different from the SMLM cases, in rFRC calculation, we generate two frames required by repetitively measuring two raw images and reconstructing them individually.

#### Comparisons of different assessments by simulation

**Fig. 5:**
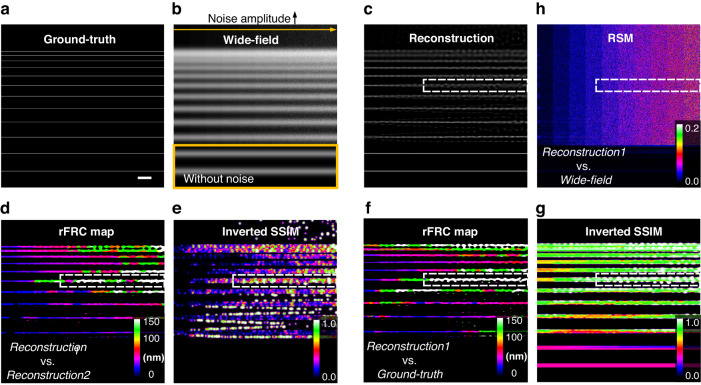
Synthesizing noise of different amplitudes to evaluate the performance of the rFRC, RSM, and SSIM. **a** Ground-truth sample. **b** Wide-field image. **c** RL deconvolution result. **d** rFRC map from two reconstructions (*Reconstruction1* vs. *Reconstruction2*). **e** Inverted SSIM map from two reconstructions (*Reconstruction1* vs. *Reconstruction2*). **f** FRC map from reconstruction and ground truth (*Reconstruction1* vs. *Ground Truth*). **g** Inverted SSIM map from reconstruction and ground truth (*Reconstruction1* vs. *Ground-truth*). **h** RSM of (**c**). Scale bar: 500 nm

First, we intend to demonstrate the reliability and superiority of our method in evaluating the image quality against other existing approaches. As seen in Fig. 5a, a series of filaments with different distances were convoluted with a wide-field PSF (numerical aperture as 1.4). We gradually increased the noise level in the raw image (along the yellow arrow, Fig. 5b) and showed the results after Richardson-Lucy (RL) deconvolution^29,30^ (Fig. 5c), which has been widely applied to improve the resolution and contrast of raw images. Compared to the ground truth, significant artifacts appeared in the white dashed box in Fig. 5c, which were successfully detected by our rFRC map (Fig. 5d) but not the RSM (Fig. 5h). On the other hand, it was found that the spatial mapping approaches induced a strong false negative, making the true negative difficult to dissect or even invisible (Fig. 5e). Under the identical configurations of rFRC mapping, we can see the structural similarity (SSIM)^47^ map, failed to highlight such unreliable regions. Beyond that, it is also worth noting that the rFRC maps formed by *Reconstruction1 and Reconstruction2* (Fig. 5d) or *Reconstruction1 and Ground-truth* (Fig. 5f) matched perfectly, indicating that our assessment can evaluate the reconstructed image quality without the ground truth.

Beyond that, our rFRC map can also be used as a generalized metric to quantify the difference between the reconstruction and ground-truth, and overcomes the natural defects of SSIM. In the SSIM map (Fig. 5g), we can see the existence of strong false negatives, making the true negatives difficult to dissect. The region inside the yellow box (Fig. 5b) was set as noise-free, thus there was no difference between the two independent reconstructions (Fig. 5d, e). Here, the reconstruction within this region (Fig. 5c) was almost identical to the ground truth (Fig. 5a). Interestingly, both the inverted SSIM map and RSM (Fig. 5h, g) still provided small values, indicating false negative. In contrast, the rFRC map between *Reconstruction1* and *Ground-truth* (Fig. 5f) remains empty for this region, which is fairly more reasonable.

#### Sensitively comparing different SIM reconstructions

**Fig. 6:**
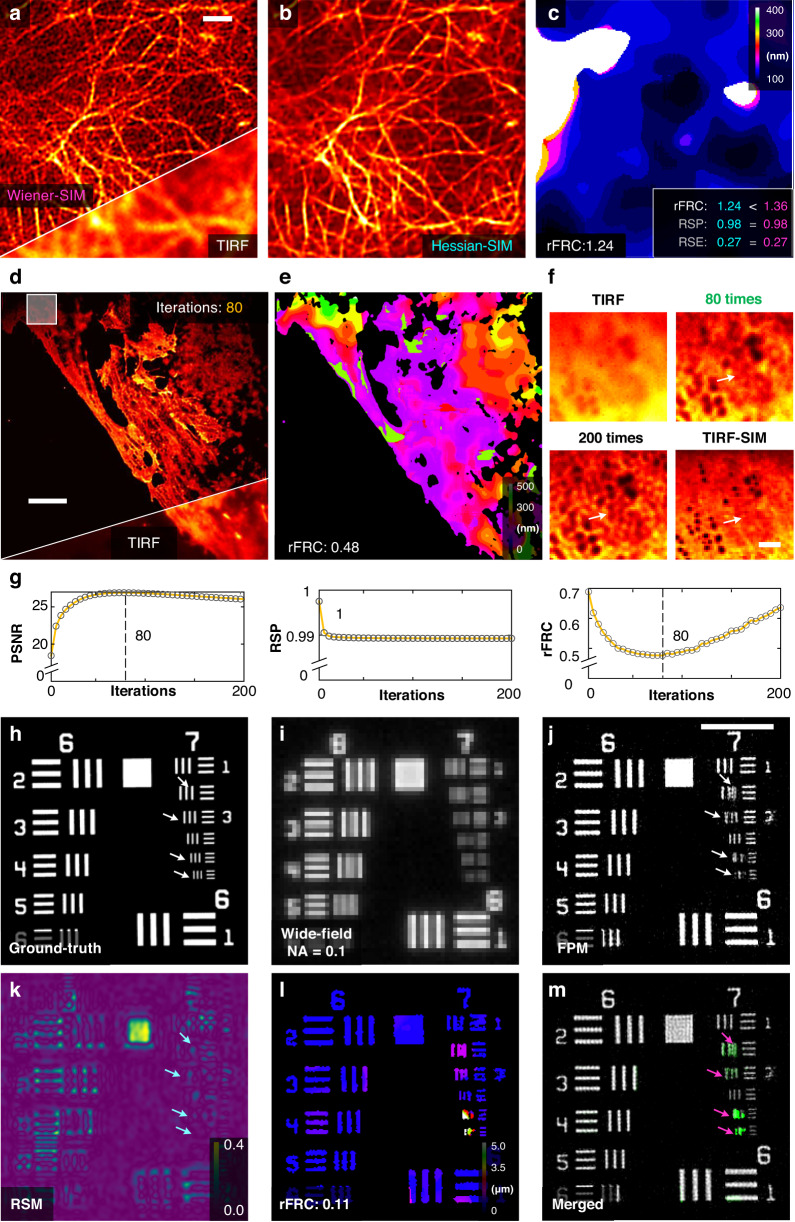
Diverse optical imaging approaches assisted and evaluated by the rFRC map. **a** Representative images of live human umbilical vein endothelial cells (HUVECs) labeled with LifeAct-EGFP under Wiener-SIM (top) and TIRF (bottom) imaging. **b** Hessian-SIM result. **c** rFRC map of Hessian-SIM. The rFRC, RSP, and RSE values of Wiener-SIM (magenta) and Hessian-SIM (cyan) are shown on the bottom right. **d** Representative results of fixed liver sinusoidal endothelial cells (LSECs) labeled with DiI under RL deconvolution (top) and TIRF (bottom) imaging. **e** rFRC map of RL deconvolution result. **f** Magnified views of the white box in (**d**). The original TIRF image, RL deconvolution results with 80 and 200 iterations, and TIRF-SIM results are shown in the top left, top right, bottom left, and bottom right, respectively. **g** Curves of the PSNR (versus TIRF-SIM), RSP (versus TIRF), and rFRC values over iterations. **h** Simulated ground-truth. **i** Wide-field image of (**h**). **j** Corresponding Fourier ptychographic microscopy (FPM) reconstruction. **k** RSM of FPM. **l** rFRC map of FPM. **m** Merged image of PANEL (green channel) and FPM (gray channel) results. Cyan and magenta arrows represent the false positives and the true negatives, respectively. Scale bars: (**a**) 1 μm; (**d**) 5 μm; (**f**) 100 nm; (**j**) 50 μm

In SIM, frequency information is unmixed and stitched from noisy data to achieve SR. As a result, its reconstruction is essentially an ill-posed inverse problem, in which the conventional Wiener reconstruction (Wiener-SIM) will amplify the noise, leading to significant fluctuations in high-frequency components. To moderate this issue, several regularizations were proposed to constrain the reconstruction^48^. For instance, the Hessian-SIM used the Hessian matrix continuity to eliminate random and non-continuous artifacts^48^. In experiments, we applied the Hessian denoising algorithm^48^ on the Wiener-SIM reconstruction^49^ (Fig. 6a) to obtain the Hessian-SIM images (Methods, Fig. 6b). Then, we performed the rFRC map to differentiate such subtle differences in the fidelity of conventional Wiener-SIM^49^ versus Hessian-SIM^48^ (rFRC value: 1.36 versus 1.24) (Fig. 6c), and in contrast, the RSM detected identical qualities (RSE value: 0.27 versus 0.27) (Supplementary Fig. S10e). It is found that only the rFRC value can reflect the difference between Wiener-SIM and Hessian-SIM. We also found that the local qualities in SIM are correlated to the emission intensity of the fluorescent signals (Supplementary Fig. S10d), in which the raw images of low SNRs are susceptible to artifacts. The unreliable regions pointed by PANEL (Supplementary Fig. S10f) are correlated to the regions under weak illumination of TIRF. Notably, the fixed pattern artifacts of SIM caused by biased parameter estimations or configurations (model bias)^12^ cannot be detected by our rFRC method.

#### Determining the number of iterations by rFRC

The traditional RL algorithm risks amplifying the noise when performing excessive iterations, which extremely limits its applications. Although the noise-insulated low-frequency components may stand stable, the maximum likelihood estimation fits the noise-dominated high-frequency ones to recover the high spatial frequencies, which will lead to wide fluctuations. As an iterative process, RL deconvolution progressively recovers the high-frequency information step by step, and in this process, the noise proportion is also increasing. Thus, the optimal iteration can protect the enhancement of high-frequency information before amplifying the noise. The common usage requires a *post hoc* visual inspection to determine the best number of iterations. Here, to ascertain the rFRC value readouts guiding this determination for the number of RL iterations, we applied RL to process the TIRF image (Fig. 6d) and then calculated its corresponding rFRC value of each iteration (Fig. 6e, right panel of Fig. 6g, see also another example in Supplementary Fig. S12). Interestingly, it is noticeable that rFRC values presented a quadratic distribution with the minimum value appearing after 80 iterations. It is similar to the peak signal-to-noise ratio distribution (PSNR, left panel of Fig. 6g), in which the TIRF-SIM (Methods) image is used as ground truth. In contrast, the curve of the resolution-scaled Pearson coefficient (RSP)^27^ failed to recapitulate this distribution (middle panel of Fig. 5g). As demonstrated in Fig. 6f, the RL deconvolution with 200 iterations produced snowflake-like artifacts, as indicated by the white arrows, which can be confirmed as nonexistent by the referenced TIRF-SIM image. A comprehensive comparison demonstrated that 80-iteration RL optimally enhanced the image contrast with the slightest noise-amplification-induced artifacts. The inverted illumination intensity map is proportional to the rFRC map (Supplementary Fig. S11), indicating that the local quality in the results of RL deconvolution is highly correlated with the SNR.

#### Extended application to coherent imaging

Fourier ptychographic microscopy (FPM)^50^ achieves high resolution by iteratively stitching together a number of low-resolution images in Fourier space, and it is a coherent imaging modality through a combination of synthetic aperture and phase retrieval concepts. In the reconstruction process, it updates the objective function between the spatial and Fourier domains iteratively with intensity or pupil constraints. In this case, the noise-contaminated high-frequency components can significantly induce quality degradation during its spectrum extension. Here, we extended our rFRC applications to FPM to assess its reconstruction qualities. The United States Air Force (USAF) resolution target was used as the ground-truth sample (Fig. 6h), and we simulated the FPM imaging process (Methods) to create the low-resolution result (Fig. 6i) and its corresponding high-resolution FPM reconstruction (Fig. 6j). In Fig. 6k, it can be seen that the RSM without filtering is prone to small intensity fluctuations belonging to false negative (FP, cyan arrows). In contrast, the rFRC map (Fig. 6l) accurately represents the quality of FPM reconstruction, pinpointing all the regions of true negative (TN, magenta arrows in Fig. 6m).

## Discussion

Without a reference in any form or its equivalent, a map of uncertainty down to the SR scale will be crucial for extracting reliable and quantitative information from biological images (Supplementary Fig. S17). According to the underlying theories of different modalities, the corresponding model bias can be minimized by optical system calibrations^12,34,40^. On the other hand, data uncertainty is fundamentally inevitable and difficult to remove, and so far, there is still no effective method for its routine evaluation. In this work, we demonstrate that the rFRC framework with two independent captures can measure the data uncertainty in general (Supplementary Note 3). When the spatially different uncertainties revealed by rFRC, the way may be paved for these state-of-the-art imaging methods to be widely adopted in cell biological studies. Assisted by rFRC, we anticipate the developers and users can optimize the resolution heterogeneity and evaluate the performances for specific experiments. Particularly, based on our analysis (Figs. 2–4), we uncover that the resolution heterogeneity in localization microscopy can be a sought-after issue to be discussed in future methodological developments and even biological studies. We expect our rFRC can be broadly used as a cross-modality tool, evaluating the resolution heterogeneity for other localization-based^22,51–53^ and fluctuation-based^54^ microscopies, offering well-founded systemic improvement schemes.

Beyond the reference-free objective quality rating, we expect our rFRC map would become a generalized metric and quality map in the presence of ground-truth, similar to the structural similarity (SSIM)^47^, to assess image quality closer to the human perception. Furthermore, as a model-independent method, our rFRC can also be applied to the recently emerged learning-based SR reconstructions^55^. By developing an open-source ImageJ plug-in, and libraries in different programming languages, we enable wide users to apply our method. We hope this metric could benefit image-based biological profiling and inspire further advances in the rapidly developing field of computational microscopes.

## Materials and methods

### FRC calculation

The FRC method measures the statistical correlation between two bidimensional signals over a series of concentric rings in the Fourier domain. It can be regarded as a function of the spatial frequency *q*_*i*_:

1 $$FR{C}_{12}({q}_{i})=\frac{\sum\nolimits _{r\in {q}_{i}}{{\mathscr{F}}}_{1}(r)\cdot {{{\mathscr{F}}}_{2}}^{\ast }(r)}{\sqrt{\sum\nolimits _{r\in {q}_{i}}{|{{\mathscr{F}}}_{1}(r)|}^{2}\cdot \sum\nolimits _{r\in {q}_{i}}{|{{\mathscr{F}}}_{2}(r)|}^{2}}}$$

where ${{\mathscr{F}}}_{1}$ and ${{\mathscr{F}}}_{2}$ denote the discrete Fourier transforms (DFTs) of the two signals and $\sum\nolimits _{r\in {q}_{i}}$ represents the summation over the pixels on the perimeter of circles of corresponding spatial frequency *q*_*i*_.

Before calculation, a Hanning window is used to suppress the edge effects and other spurious correlations caused by the DFT calculation. The rectangular images should be zero-padded to produce squares to calculate the FRC curve. To calculate the discrete values of the corresponding spatial frequencies, it is necessary to define the discretization of the spatial frequencies of the FRC curve. The maximum frequency *f*_max_ is half the inverse of the pixel size (*p*_*s*_), i.e., *f*_max_ = 1/(2 *p*_*s*_). Then the average filter with a half-width of the average window (equal to 3 frequency bins) is applied to smooth this noisy FRC curve.

When the FRC curve drops below a given threshold, the corresponding frequency is defined as the effective cutoff frequency (COF), whereas the resolution is the inverse of the effective COF. This threshold for FRC indicates the spatial frequency above which meaningful information beyond random noise can be extracted. Specifically, the common choices for the criterion/threshold are the fixed-value thresholds or the sigma-factor curves^36^. The fixed value is usually the 1/7 hard threshold, and the criterion of sigma-factor curves can be written as follows:

2 $${\sigma }_{i}=\frac{{\sigma }_{factor}}{\sqrt{{N}_{i}/2}}$$

where *N*_*i*_ represents the number of pixels in a ring of radius *q*_*i*_ and the most commonly used *σ*_*factor*_ is 3. If the two measurements are corrupted with excessive noise, the FRC curve can be expressed as $FR{C}_{i}=1/\sqrt{{N}_{i}}$^36^.

The 1/7 hard threshold has been widely used in determining the resolution of SR images. Although this fixed-value threshold method is incompatible with statistical assumptions^36^, the resolution obtained with that criterion is approximately accurate for SMLM^24^ and the stimulated emission depletion microscopy (STED) microscopy^56^. The 1/7 threshold attains a similar result for a large image to the 3*σ* curve criterion (Supplementary Fig. S15a). However, this fixed threshold is overconfident for determining the resolutions of small image blocks, which is essential to map local SR errors in the reconstructions. In Supplementary Fig. S15a, the 1/7 threshold is smaller than all correlation values in the FRC curve and fails to yield the COF of small images (red cross). On the other hand, unlike avoiding the conservative threshold choice in resolution determination, we prefer a moderate threshold for quality mapping to reduce false positives. Therefore, we choose three standard deviations above the expected random noise fluctuations as the threshold^36^. This criterion is robust and accurate in examining small image blocks and calculating the FRC resolutions.

### rFRC map generation

#### Two-frame generation

The rFRC mapping requires two independent frames of identical contents under the same imaging conditions. For the SMLM and the SRRF modalities (Figs. 2–4, Supplementary Figs. S6-S9), these two frames were generated by splitting the raw image sequence in half (odd and even frames) and reconstructing the resulting two image subsets independently. For the SIM, FPM, and RL deconvolution (Fig. 6, Supplementary Figs. S10-S12), we directly imaged the identical contents twice to capture the required two frames.

#### rFRC mapping

Since the FRC measures the global similarity between two images, we extend the FRC to a rolling form (rFRC) to provide the local distance measurements at the pixel level. We regard the FRC calculations as a filter in which the image is scanned block by block (64 × 64 pixels as a default size in this work), with each block assigned the corresponding FRC resolution. First, we pad the input image symmetrically around a half size of the block to calculate the FRC at the image boundaries (Step 1, Fig. 1a). Second, by setting the background threshold of the center pixel, we avoid FRC calculation of the background area. If the mean of the center pixels is larger than the threshold, we calculate the FRC and assign the FRC resolution to the center pixel of each block. In contrast, we set a zero value to the central pixel when it is smaller than the threshold (Steps 2–4, Fig. 1a). Afterward, we run this procedure block by block until the entire image is finished.

#### Background thresholding

By labeling designated structures specifically, fluorescence images confer high contrast and dark background areas containing background and readout noise. These regions, however, result in low FRC resolutions that are essentially false negatives. Therefore, we use two strategies to threshold the background (Supplementary Fig. S2). We determine the hard threshold according to the images by user-defined global value adapting to their data (default method) or by an iterative wavelet transform method^57^ to estimate local values automatically. For the global threshold, because different values lead to different regions being interrogated, we choose the hard threshold carefully based on two principles: (1) the removal of background; (2) the maintenance of structures. Regarding the local threshold, the background is iteratively estimated from the lowest-frequency wavelet bands of the images (Supplementary Fig. S2c). In each iteration, all image values above the current estimation are clipped.

#### rFRC mapping acceleration

Although the rFRC allows evaluation at the pixel level, the most delicate scale of detectable errors can only reach the highest resolution allowed by the system, which satisfies the Nyquist-Shannon sampling theorem. Thus, the smallest error should be larger than ~3 × 3 pixels. Therefore, we can skip 2~4 pixels for each rolling operation to accelerate the mapping calculation 4~16 times. The rFRC map can be resized to the original image size by bilinear interpolation for better visualization.

#### Adaptively filtering the rFRC map

The FRC calculation is not always stable and may generate aberrantly large values in neighboring pixels due to improperly determined COFs. Thus, we create an adaptive median filter to remove these inappropriate values. Instead of the standard median filter that replaces each pixel with the median of the neighboring pixels, we develop an adaptive median filter to remove only the isolated pixels with aberrantly large values, avoiding blurring of the rFRC map^15^. If the pixel intensity is larger than a preset fold (default as 2-fold) of the median in the window (default as 3-pixel), the pixel is replaced by the median value. Otherwise, the window moves to the next pixel.

#### Drift correcting

To correct relative movements between measurements, we use a method based on the phase correlation^58^. First, we calculate the cross-correlation function *CC* of the two images:

3 $$CC(x,y)=\tilde{{\mathscr{F}}}\{{\mathscr{F}}({M}_{1})\cdot {\mathscr{F}}{({M}_{2})}^{\ast }\}$$

where *M*_1_ and *M*_2_ represent the two images. The peak of the *CC* is the shift between these two images that ensures the best-correlated *M*_1_ and *M*_2_. After that, we find the centroid of the distribution of intensities of the cross-correlation function to achieve subpixel accuracy. This operation is executed before the rFRC mapping.

### rFRC colormap

Choosing a proper color map to visualize error maps is another tricky question. The existing popular color maps, such as Jet, use blue to red to index the different error magnitudes. However, people usually tend to define black (dark color) as small magnitude and white (light color) as large magnitude, which is identical to the logic of the gray color map. In this sense, the Jet color map may be incompatible with human intuition^37^. On the other hand, human vision is insensitive to light or dark gray levels and sensitive to different colors. As a result, we intend to create a color map using color to index the magnitudes and with black/white zone to visualize the smallest/largest values.

First, because human eyes are more sensitive to green color, we use green to highlight errors of large magnitude. Second, human instinct usually regards bright color (white) as an effect of large magnitude and dark color (black) for small magnitudes. Therefore, we involve a black zone (0, 0, 0) and a white zone (1, 1, 1) in the color map to visualize the smallest and largest values. Taken together, we shift the Jet colormap (left panel of Supplementary Fig. S3a) to create the shifted Jet (sJet) color map (right panel of Supplementary Fig. S3a). Along with the extension of the blue color component in this sJet color map, we obtain a white zone to represent the most significant error (even larger than those highlighted in green). Because the background in the rFRC map means no error, we use the black zone for the display. As shown in Supplementary Fig. S3a, our sJet color map is more intuitive for visualizing errors than the original Jet color map.

In addition to the sJet colormap, we also provided another alternative colormap, i.e., Jet with the black zone (bJet, middle panel of Supplementary Fig. S3a) while using red color to represent large magnitude. The readers are encouraged to try these colormaps and select their favorite ones.

### rFRC value

As mentioned above, the rFRC map can be used to subtly visualize the local uncertainties down to the SR scale. Here, we also intend to give two metrics for globally evaluating the entire image quality. One metric with dimension (*resolution*) represented the averaged resolution across the entire imaging field, namely rFRC resolution, and its calculation is given as follows:

4 $$\frac{\sum\nolimits_{FV\ne 0}FV(x,y)}{{\Vert FV(x,y)\Vert }_{0}}$$

where ${\Vert FV\Vert }_{0}$ is the *l*_*0*_ norm, which represents the number of nonzero values in the rFRC map, and *FV* denotes the rFRC map.

Secondly, to reflect the potential deterioration rate of the reconstructed images, we provided a more generalized dimensionless metric, namely rFRC value. Here we normalize the rFRC resolution with its corresponding minimum resolution, and subtract 1 to ensure its value starting at 0:

5 $$\frac{\sum\nolimits_{FV\ne 0}FV(x,y)}{{\Vert FV(x,y)\Vert }_{0}\cdot \,\min (FV(x,y))}-1$$

It noted that both metrics can be further extended to three dimensions, in which the (*x*, *y*) two-dimensional coordinates can be raised to three dimensions (*x*, *y*, *z*) directly (3D rFRC value).

### RSM generation

#### Image intensity rescaling and resolution scaling function (RSF) estimation

To normalize the intensity between low-resolution (LR) and high-resolution (HR) images and maximize the similarity between them, the intensity of the original HR image *I*_*H*_ needs to be linearly rescaled:

6 $${I}_{HS}(\mu ,\theta )={I}_{H}\times \mu +\theta$$

where *I*_*HS*_ represents the HR image after linear rescaling. The values of *μ* and *θ* in Eq. (6) should be chosen to maximize the similarity between the LR image, *I*_*L*_, and *I*_*HS*_ convolved with the RSF. Because the RSF is an unknown kernel used to transform an HR image into an LR image, it can be approximatively defined by a 2D Gaussian function with an unknown *σ*. The RSF is usually anisotropic in the *x* and *y* directions. Hence unlike its original version^27^, we set *σ* as a vector that includes two elements, i.e., *σ*_*x*_ and *σ*_*y*_.

Then, to estimate *μ* and *θ* for image intensity rescaling and *σ*_*x*_ and *σ*_*y*_ for RSF parameterization, we jointly optimize these four variables (Supplementary Fig. S4), i.e., *μ*, *θ*, *σ*_*x*_, and *σ*_*y*_, to minimize the following function:

7 $$\mathop{{\rm{arg}}\,\min}\limits_{\mu ,\theta ,{\sigma }_{x},{\sigma }_{y}}{\Big\Vert {I}_{L}-{I}_{HS}(\mu ,\theta )\otimes {I}_{RSF}({\sigma }_{x},{\sigma }_{y})\Big\Vert }_{2}^{2}$$

Because the gradient in Eq. (7) is difficult to calculate, we use a derivative-free optimizer to search for the four optimal parameters. Different from the particle swarm optimization (PSO)^59^ used previously^27^, we chose the pattern search method (PSM)^60^ to optimize Eq. (7). PSO searches for substantial candidate solutions and may be not necessary for a four-parameter optimization problem. Compared to the unstable and slow metaheuristic optimization approach of PSO, the PSM is stable, computationally effective, and direct. It is commonly used in small-scale parameter optimization problems and is more suitable for our RSM estimation.

### Metrics and pixel-wise error map of the RSM

After obtaining *μ* and *θ* (image intensity rescaling factors) and *σ*_*x*_ and *σ*_*y*_ (RSF parameters), we can transform the HR image *I*_*H*_ into its LR version *I*_*HL*_ by convolving the estimated RSF.

8 $${I}_{HL}=({I}_{L}\times \mu +\theta )\otimes RSF={I}_{HS}(\mu ,\theta )\otimes {I}_{RSF}(\sigma )$$

To assess the global quality of the resolution-scaled-back image *I*_*HL*_ against the original LR image *I*_*L*_, we use the common root mean squared error for the resolution-scaled error (RSE)^27^ and the Pearson correlation coefficient for the resolution-scaled Pearson coefficient (RSP)^27^.

9 $$\begin{array}{c}{\rm{RSE}}=\sqrt{\frac{{\sum }_{x,y}{I}_{L}(x,y)-{I}_{HL}{(x,y)}^{2}}{n}}\\\qquad\quad {\rm{RSP}}=\frac{{\sum }_{x,y}({I}_{L}(x,y)-{\bar{I}}_{L})({I}_{HL}-{\bar{I}}_{HL})}{\sqrt{{\sum }_{x,y}{({I}_{L}-{\bar{I}}_{L})}^{2}}\sqrt{{\sum }_{x,y}{({I}_{HL}-{\bar{I}}_{HL})}^{2}}}\end{array}$$

In addition, to visualize the pixelwise absolute difference, the RSM between *I*_*L*_ and *I*_*HL*_ can be calculated by:

10 $${\rm{RSM}}({\rm{x}},{\rm{y}})=|{I}_{L}(x,y)-{I}_{HL}(x,y)|$$

### PANEL pinpointing

To pinpoint regions with a high probability of error existence, we filter both the RSM and the rFRC to create a PANEL composite map. The small-magnitude components contained in the RSM may introduce false negatives. Therefore, we segment the RSM before integrating it into PANEL by the following equation:

11 $$\tilde{R}(x,y)=\left\{\begin{array}{l}R(x,y),\,R(x,y)\in [0.5,1]\\ 0,\,R(x,y)\in [0,0.5)\end{array}\right.$$

where *R*(*x*, *y*) represents the normalized RSM value in the *x*, *y* positions and $\tilde{R}$ denotes the segmented RSM. After this operation, the small false negative is filtered, leaving us with strong low-resolution scale error components, focusing on the true negatives detected by the RSM. On the other hand, the rFRC map indicates the degree of uncertainty. The smallest FRC value in the map may not represent the error existence. Likewise, we introduce a segmentation method called Otsu^38^, which automatically determines the threshold by maximizing the interclass variance, performing image thresholding to filter the background in the rFRC map, and highlighting the regions with a high possibility of error existence (Supplementary Fig. S3b).

After that, considering human eyes more sensitive to the green color, we used the rFRC as green channel for better visualization of fine details, and leave the red channel for RSM to display large-scale components. In detail, first, the rFRC map and the RSM are normalized to a 0~1 scale. Second, we filter the rFRC map and the RSM with the ‘Otsu determined threshold’ and the ‘0.5 threshold’, respectively. Regions with values smaller than the thresholds are set to zero, and regions with larger values remain unchanged. Finally, we merge the rFRC map (green channel) and the RSM (red channel), and this operation is for qualitative pinpointing of regions with low reliability. The original rFRC map and the 0.5 threshold filtered RSM can be separated if quantitative evaluations are required.

In addition, if the datasets are three-dimensional or under a non-Gaussian convolution relation (between the low-resolution and high-resolution scales), we cannot estimate the corresponding RSMs. For these datasets, the RSM is not integrated into PANEL.

### SMLM Fusion

The RSM estimates the errors at the low-resolution scale, which is not suitable for the SMLM fusion. In contrast, the rFRC estimates the degree of errors at the SR scale and thus is a superior choice to guide the fusion of SMLM. Using the rFRC quality metric, we can fuse different localization results according to the weights of the rFRC maps, resulting in combined reconstructions that perform better than any one of the reconstructions alone.

12 $$\frac{\mathop{\sum }\nolimits_{n=1}^{N}{L}_{n}\cdot \{G(\sigma )\otimes (\max ({F}_{1 \sim N})-{F}_{n})\}}{\mathop{\sum }\nolimits_{n=1}^{N}G(\sigma )\otimes (\max ({F}_{1 \sim N})-{F}_{n})}$$

where *L*_*n*_ is the result of the *n*^*th*^ localization model, and *G*(*σ*) represents the Gaussian kernel with *σ* standard variance. The max(*F*_*1~N*_) is the maximum FRC value of the total *N* localization results, and ⊗ is the convolution operation. We use *G* (*σ* as 4 pixels) to slightly blur the rFRC map, avoiding oversharpen effects.

### STORM imaging

#### Microscope setup

After washing with phosphate buffer saline (PBS), the samples were mounted on glass slides with a standard STORM imaging buffer consisting of 5% w/v glucose, 100 × 10^−3^_M_ cysteamine, 0.8 mg mL^−1^ glucose oxidase, and 40 µg mL^−1^ catalase in Tris-HCl (pH 7.5)^43^. Then, data were collected by 3D-STORM^43^ carried out on a homebuilt setup based on a modified commercial inverted fluorescence microscope (Eclipse Ti-E, Nikon) using an oil-immersion objective (100×/1.45 NA, CFI Plan Apochromat λ, Nikon). Lasers at 405 nm and 647 nm were introduced into the cell sample through the objective’s back focal plane and shifted toward the edge of the objective to illuminate ~1 µm within the glass-water interface. A strong (~2 kW cm^−2^) excitation laser of 647 nm photoswitched most of the labeled dye molecules into a dark state while also exciting fluorescence from the remaining sparsely distributed emitting dye molecules for single-molecule localization. A weak (typical range: 0–1 W cm^−2^) 405 nm laser was used concurrently with the 647 nm laser to reactivate fluorophores into the emitting state. Only a small, optically resolvable fraction of fluorophores was emitting at any given instant. A cylindrical lens was put into the imaging path to introduce astigmatism to encode the depth (z) position into the ellipticity of the single-molecule images^43^. The EMCCD (iXon Ultra 897, Andor) camera recorded images at a 110-frame-rate for a frame size of 256 × 256 pixels and typically recorded ≈50,000 frames for each experiment. In addition, to form the 2D-STORM imaging, we removed the cylindrical lens in the optical layout.

#### STORM reconstruction

The open-source software package Thunder-STORM^41^ and customized 3D-STORM software^43^ were used for STORM image reconstruction. Images labeled ‘ME-MLE’ and ‘SE-MLE’ were reconstructed by Thunder-STORM with maximum likelihood estimation (integrated PSF method), and multi-emitter fitting enabled (ME-MLE) or not (SE-MLE). The images labeled ‘SE-Gaussian’ were reconstructed with the customized 3D-STORM software by fitting local maxima with an (elliptical) Gaussian function described previously in ref. ^43^. Drift correction was performed post-localization, and images were rendered using a normalized Gaussian function (σ as 2 pixels).

#### Cell culture, fixation, and immunofluorescence

COS-7 cells were cultured in DMEM (GIBCO, 21063029) supplemented with 10% fetal bovine serum (FBS; GIBCO) in a humidified CO_2_ incubator with 5% CO_2_ at 37 °C, following standard tissue-culture protocols. Then, cells were seeded on 12 mm glass coverslips in a 24-well plate at ~2 × 10^4^ cells per well and cultured for 12 h. For STORM of actin filaments, a previously established fixation protocol^61^ was employed: The samples were first fixed and extracted for 1 min with 0.3% v/v glutaraldehyde and 0.25% v/v Triton X-100 in cytoskeleton buffer (CB, 10 × 10^−3^_M_ MES, pH 6.1, 150 × 10^−3^_M_ NaCl, 5 × 10^−3^_M_ EGTA, 5 × 10^−3^_M_ glucose, and 5 × 10^−3^_M_ MgCl_2_), postfixed for 15 min in 2% (v/v) glutaraldehyde in CB, and reduced with a freshly prepared 0.1% sodium borohydride solution in PBS. Alexa Fluor 647-conjugated phalloidin was applied at a concentration of ≈0.4 × 10^−6^_M_ for 1 h. The sample was briefly washed two to three times with PBS and then immediately mounted for imaging. For the imaging of other targets, samples were fixed with 3% w/v paraformaldehyde and 0.1% w/v glutaraldehyde in PBS for 20 min. After reduction to a freshly prepared 0.1% sodium borohydride solution in PBS for 5 min, the samples were permeabilized and blocked in blocking buffer (3% w/v BSA, 0.5% v/v Triton X-100 in PBS) for 20 min. Afterward, the cells were incubated with the primary antibody (described above) in a blocking buffer for 1 h. After washing in a washing buffer (0.2% w/v BSA and 0.1% v/v Triton X-100 in PBS) three times, the cells were incubated with the secondary antibody for 1 h at room temperature. Then, the samples were washed three times with the washing buffer before being mounted for imaging.

### SIM imaging

#### TIRF-SIM

Our SIM system was built upon a commercial inverted fluorescence microscope (IX83, Olympus) equipped with a TIRF objective (100×/1.7 NA, Apo N, HI Oil, Olympus) and a multiband dichroic mirror (DM, ZT405/488/561/640-phase R; Chroma) as described previously^48^. In short, laser light with wavelengths of 488 nm (Sapphire 488LP-200) and 561 nm (Sapphire 561LP-200, Coherent) and acoustic, optical tunable filters (AOTFs, AA Opto-Electronic, France) were used to combine, switch, and adjust the illumination power of the lasers. A collimating lens (focal length: 10 mm, Lightpath) was used to couple the lasers to a polarization-maintaining single-mode fiber (QPMJ-3AF3S, Oz Optics). The output lasers were then collimated by an objective lens (CFI Plan Apochromat Lambda 2× NA 0.10, Nikon) and diffracted by a pure phase grating that consisted of a polarizing beam splitter, a half-wave plate, and an SLM (3DM-SXGA, ForthDD). The diffraction beams were then focused by another achromatic lens (AC508-250, Thorlabs) onto the intermediate pupil plane, where a carefully designed stop mask was placed to block the zero-order beam and other stray light and to permit passage of ±1 ordered beam pairs only. To maximally modulate the illumination pattern while eliminating the switching time between different excitation polarizations, a homemade polarization rotator was placed after the stop mask. Next, the light passed through another lens (AC254-125, Thorlabs) and a tube lens (ITL200, Thorlabs) to be focused onto the back focal plane of the objective lens, interfering with the image plane after passing through the objective lens. Emitted fluorescence collected by the same objective passed through a dichroic mirror, an emission filter, and another tube lens. Finally, the emitted fluorescence was split by an image splitter (W-VIEW GEMINI, Hamamatsu, Japan) before being captured by a sCMOS (Flash 4.0 V3, Hamamatsu, Japan) camera.

#### Hessian-SIM

We applied the Hessian denoising algorithm^48^ without the *t* continuity constraint on the Wiener-SIM reconstruction^49^ results to obtain the Hessian-SIM images, as shown in Fig. 6b.

#### Cell maintenance and preparation

Human umbilical vein endothelial cells (HUVECs) were isolated and cultured in an M199 medium (Thermo Fisher Scientific, 31100035) supplemented with fibroblast growth factor, heparin, and 20% FBS or in an endothelial cell medium (ECM) (ScienCell, 1001) containing endothelial cell growth supplement (ECGS) and 10% FBS. The cells were infected with a retrovirus system to express LifeAct-EGFP. The transfected cells were cultured for 24 h, detached using trypsin-EDTA, seeded onto poly-_L_-lysine-coated coverslips (H-LAF10L glass, reflection index: 1.788, thickness: 0.15 mm, customized), and cultured in an incubator at 37 °C with 5% CO_2_ for an additional 20–28 h before the experiments. Liver sinusoidal endothelial cells (LSECs) were isolated and plated onto 100 µg/ml collagen-coated coverslips and cultured in high-glucose DMEM supplemented with 10% FBS, 1% _L_-glutamine, 50 U/ml penicillin, and 50 µg/ml streptomycin in an incubator at 37 °C with 5% CO_2_ for 6 h before imaging. Live cells were incubated with DiI (100 µg/ml, Biotium, 60010) for 15 min at 37 °C, whereas fixed cells were fixed with 4% formaldehyde at room temperature for 15 min before labeling with DiI. For the SIM imaging experiments, cells were seeded onto coverslips (H-LAF 10 L glass, reflection index: 1.788, diameter: 26 mm, thickness: 0.15 mm, customized).

### Open-source datasets

In addition to the custom-collected datasets, we also used freely available simulation/experiment datasets to illustrate the broad applicability of our method.

#### 2D-SMLM simulation datasets

The ‘*Bundled Tubes High Density*’ (361 frames) and ‘*Bundled Tubes Long Sequence*’ (12000 frames) datasets from the ‘*Localization Microscopy Challenge datasets*'^13^ on the EPFL website were used as the high-density and low-density 2D-SMLM simulation datasets in this work, as shown in Fig. 2a. The NA of the optical system was 1.4 (oil-immersion objective), and the wavelength of the fluorescence was 723 nm.

#### 3D-SMLM simulation datasets

The ‘*MT1.N1.LD*’ (19996 frames, 3D-Astigmatism PSF) dataset from the ‘*Localization Microscopy Challenge datasets*'^42^ on the EPFL website was used as the low-density 3D-SMLM simulation dataset in this work, as shown in Supplementary Fig. S6. The NA of the optical system was 1.49 (oil-immersion objective), and the wavelength of the fluorescence was 660 nm. All the images had a frame size of 64 × 64 pixels (pixel size as 100 nm). Then, 20 frames from this low-density dataset were averaged into one frame to generate the corresponding high-density 3D-SMLM dataset (resulting in 998 frames).

#### 2D-SMLM experimental datasets

The ‘*Localization Microscopy Challenge datasets*'^13^ also contain experimental data, and 500 high-density images of tubulins were acquired from the EPFL website (Supplementary Fig. S7a, S7b). The NA of the optical system was 1.3 (oil-immersion objective), and the wavelength of the fluorescence was 690 nm. The images were recorded with a camera at a 25-frame-rate for a frame size of 64 × 64 pixels (pixel size as 100 nm).

#### Live-cell SRRF datasets

The GFP-tagged microtubules in live HeLa cells were imaged by the TIRF mode with a TIRF objective (100×/1.46 NA, Plan Apochromat, Oil, Zeiss) and an additional 1.6× magnification with 488 nm laser illumination^28^ (200 frames in total). The open-source ImageJ plugin^28^ was used to reconstruct the SRRF results (Supplementary Fig. S7c).

### Simulations of the grid imaged by SMLM

Following ref. ^27^, we created a regular grid on a pixel of 10 nm in size (Supplementary Fig. S4c). The density of the randomly activated molecule was set as increasing gradually from the center to the sides. Then, the resulting image sequence was convoluted with a Gaussian kernel with an FWHM of 280 nm and down-sampled ten times (pixel size 100 nm). After that, Poisson and 20% Gaussian noise were injected into the image sequence (Supplementary Fig. S4d). Finally, the image sequence was reconstructed by Thunder-STORM with maximum likelihood estimation (integrated PSF method), which enabled the multi-emitter fitting function.

### Simulation of Fourier ptychographic microscopy (FPM)

We used the United States Air Force (USAF) resolution target as the ground-truth sample of the FPM^50^ (Fig. 6h). The intensity and phase of the imaged sample were both set as those of the USAF target with a size of 240 × 240 pixels (pixel size: 406.3 nm). Illumination from different angles was provided by a 7 × 7 LED matrix, whose emission wavelength was 532 nm and distance to the sample was 90 mm. The sample was illuminated by each LED unit, filtered by the objective (4×/0.1 NA), and sampled by the camera (image size as 60 × 60 and pixel size as 1.625 μm). After the LEDs illuminated the sample, the final 49 low-resolution images were obtained. We used the image illuminated by the LED in the center as the initial image. Then, the amplitude and phase of the corresponding aperture were updated in turn in each FPM iteration. After 10 iterations, the final high-resolution complex-amplitude image (240 × 240) was obtained, the size of which was enlarged by 4× compared to the corresponding low-resolution images.

### Image rendering and processing

We used the custom-developed color maps, shifted Jet and black Jet (sJet and bJet), to visualize the rFRC maps in this work. The color maps ‘SQUIRREL-FRC'^27^ were used to present the FRC maps in the Supplementary Fig. S15d, and S17. The color maps ‘SQUIRREL-Errors'^27^ were used to present the difference map in the second and third columns of Fig. 2, the bottom panel of Fig. 4f, and the third panel of Supplementary Fig. S7b. The volumes in Fig. 3d–f were rendered by ClearVolume^62^. The Jet color map projection was used to show the depth in Supplementary Fig. S8b. All data processing was achieved using MATLAB and ImageJ. All figures were prepared with MATLAB, ImageJ, Microsoft Visio, and OriginPro.

### Supplementary information

Supplementary Information

## Data Availability

All the data that support the findings of this study are available from the corresponding author on request.

## Code availability

The updated version of this work in MATLAB library can be found at https://github.com/WeisongZhao/PANELM, and the corresponding Python library can be found at https://github.com/WeisongZhao/PANELpy. The updated ImageJ plugin and its source code can be found at https://github.com/WeisongZhao/PANELJ. The online tutorials can be found on the corresponding GitHub wikis.

## References

[1] Schermelleh L (2019). Super-resolution microscopy demystified. Nat. Cell Biol..

[2] Gao P, Yuan C (2022). Resolution enhancement of digital holographic microscopy via synthetic aperture: a review. Light: Adv. Manuf..

[3] Liu Y, Zhang X, Su F, Guo Z, Jin D (2023). Contrast-enhanced fluorescence microscope by LED integrated excitation cubes. Light: Adv. Manuf..

[4] Zeng ZP (2017). Computational methods in super-resolution microscopy. Front. Inf. Technol. Electron. Eng..

[5] Dempsey GT (2011). Evaluation of fluorophores for optimal performance in localization-based super-resolution imaging. Nat. Methods.

[6] Diekmann R (2020). Optimizing imaging speed and excitation intensity for single-molecule localization microscopy. Nat. Methods.

[7] Helmerich DA (2021). Photoblueing of organic dyes can cause artifacts in super-resolution microscopy. Nat. Methods.

[8] Van De Linde S (2011). Direct stochastic optical reconstruction microscopy with standard fluorescent probes. Nat. Protoc..

[9] Almada P, Culley S, Henriques R (2015). PALM and STORM: Into large fields and high-throughput microscopy with sCMOS detectors. Methods.

[10] Erdélyi M (2015). Origin and compensation of imaging artefacts in localization-based super-resolution microscopy. Methods.

[11] Schaefer LH, Schuster D, Schaffer J (2004). Structured illumination microscopy: artefact analysis and reduction utilizing a parameter optimization approach. J. Microsc..

[12] Demmerle J (2017). Strategic and practical guidelines for successful structured illumination microscopy. Nat. Protoc..

[13] Sage D (2015). Quantitative evaluation of software packages for single-molecule localization microscopy. Nat. Methods.

[14] Mo YQ (2021). Structured illumination microscopy artefacts caused by illumination scattering. Philos. Trans. R. Soc. A: Math., Phys. Eng. Sci..

[15] Zhao WS (2022). Sparse deconvolution improves the resolution of live-cell super-resolution fluorescence microscopy. Nat. Biotechnol..

[16] Thevathasan JV (2019). Nuclear pores as versatile reference standards for quantitative superresolution microscopy. Nat. Methods.

[17] Scheckenbach M (2020). DNA origami nanorulers and emerging reference structures. APL Mater..

[18] Betzig E (2006). Imaging intracellular fluorescent proteins at nanometer resolution. Science.

[19] Luo Y (2022). Computational imaging without a computer: seeing through random diffusers at the speed of light. eLight.

[20] Marsh RJ (2021). Sub-diffraction error mapping for localisation microscopy images. Nat. Commun..

[21] Rust MJ, Bates M, Zhuang XW (2006). Sub-diffraction-limit imaging by stochastic optical reconstruction microscopy (STORM). Nat. Methods.

[22] Balzarotti F (2016). Nanometer resolution imaging and tracking of fluorescent molecules with minimal photon fluxes. Science.

[23] Ball G (2015). SIMcheck: a toolbox for successful super-resolution structured illumination microscopy. Sci. Rep..

[24] Nieuwenhuizen RPJ (2013). Measuring image resolution in optical nanoscopy. Nat. Methods.

[25] Baxter WT (2009). Determination of signal-to-noise ratios and spectral SNRs in cryo-EM low-dose imaging of molecules. J. Struct. Biol..

[26] Balzarotti F (2017). Nanometer resolution imaging and tracking of fluorescent molecules with minimal photon fluxes. Science.

[27] Culley S (2018). Quantitative mapping and minimization of super-resolution optical imaging artifacts. Nat. Methods.

[28] Gustafsson N (2016). Fast live-cell conventional fluorophore nanoscopy with ImageJ through super-resolution radial fluctuations. Nat. Commun..

[29] Richardson WH (1972). Bayesian-based iterative method of image restoration. J. Opt. Soc. Am..

[30] Lucy LB (1974). An iterative technique for the rectification of observed distributions. Astron. J..

[31] Schindelin J (2012). Fiji: an open-source platform for biological-image analysis. Nat. Methods.

[32] Kendall, A. & Gal, Y. What uncertainties do we need in bayesian deep learning for computer vision? in *Proc. 31st International Conference on Neural Information Processing Systems (NIPS)* (eds Wallach, H., Larochelle, A., Beygelzimer, F., d’Alché-Buc, Fox. & R., Garnett) 5580–5590 (The Neural Information Processing Systems Foundation, 2017).

[33] Zelger P (2021). Three-dimensional single molecule localization close to the coverslip: a comparison of methods exploiting supercritical angle fluorescence. Biomed. Opt. Express.

[34] You S (2021). Microscope calibration protocol for single-molecule microscopy. Opt. Express.

[35] Zhai GT, Min XK (2020). Perceptual image quality assessment: a survey. Sci. China Inf. Sci..

[36] Van Heel M, Schatz M (2005). Fourier shell correlation threshold criteria. J. Struct. Biol..

[37] Crameri F, Shephard GE, Heron PJ (2020). The misuse of colour in science communication. Nat. Commun..

[38] Otsu N (1979). A threshold selection method from gray-level histograms. IEEE Trans. Syst., Man, Cybern..

[39] Beker W (2020). Minimal-uncertainty prediction of general drug-likeness based on Bayesian neural networks. Nat. Mach. Intell..

[40] Faklaris O (2022). Quality assessment in light microscopy for routine use through simple tools and robust metrics. J. Cell Biol..

[41] Ovesný M (2014). ThunderSTORM: a comprehensive ImageJ plug-in for PALM and STORM data analysis and super-resolution imaging. Bioinformatics.

[42] Sage D (2019). Super-resolution fight club: Assessment of 2D and 3D single-molecule localization microscopy software. Nat. Methods.

[43] Huang B (2008). Three-dimensional super-resolution imaging by stochastic optical reconstruction microscopy. Science.

[44] Obara CJ, Moore AS, Lippincott-Schwartz J (2023). Structural diversity within the endoplasmic reticulum—from the microscale to the nanoscale. Cold Spring Harb. Perspect. Biol..

[45] Vilas JL, Tagare HD (2023). New measures of anisotropy of cryo-EM maps. Nat. Methods.

[46] Min JH (2014). FALCON: fast and unbiased reconstruction of high-density super-resolution microscopy data. Sci. Rep..

[47] Wang Z (2004). Image quality assessment: from error visibility to structural similarity. IEEE Trans. Image Process..

[48] Huang XS (2018). Fast, long-term, super-resolution imaging with Hessian structured illumination microscopy. Nat. Biotechnol..

[49] Gustafsson MGL (2008). Three-dimensional resolution doubling in wide-field fluorescence microscopy by structured illumination. Biophys. J..

[50] Zheng GA, Horstmeyer R, Yang C (2013). Wide-field, high-resolution Fourier ptychographic microscopy. Nat. Photonics.

[51] Errico C (2015). Ultrafast ultrasound localization microscopy for deep super-resolution vascular imaging. Nature.

[52] Heath GR (2021). Localization atomic force microscopy. Nature.

[53] Zhao K, Xu X, Ren W, Jin D, Xi P (2022). Two-photon MINFLUX with doubled localization precision. eLight.

[54] Zhao WS (2023). Enhanced detection of fluorescence fluctuations for high-throughput super-resolution imaging. Nat. Photonics.

[55] Yang TJ (2021). Advancing biological super-resolution microscopy through deep learning: a brief review. Biophys. Rep..

[56] Tortarolo G (2018). Evaluating image resolution in stimulated emission depletion microscopy. Optica.

[57] Galloway CM, Le Ru,EC, Etchegoin PG (2009). An iterative algorithm for background removal in spectroscopy by wavelet transforms. Appl. Spectrosc..

[58] Guizar-Sicairos M, Thurman ST, Fienup JR (2008). Efficient subpixel image registration algorithms. Opt. Lett..

[59] Poli R, Kennedy J, Blackwell T (2007). Particle swarm optimization. Swarm Intell..

[60] Lewis RM, Torczon V, Trosset MW (2000). Direct search methods: then and now. J. Comput. Appl. Math..

[61] Xu K, Babcock HP, Zhuang XW (2012). Dual-objective STORM reveals three-dimensional filament organization in the actin cytoskeleton. Nat. Methods.

[62] Royer LA (2015). ClearVolume: open-source live 3D visualization for light-sheet microscopy. Nat. Methods.

