# Supplementary material for: Quantitatively mapping local quality of super-resolution microscopy by rolling Fourier ring correlation
Source: Light Sci Appl. 2023 Dec 14;12:298. doi: 10.1038/s41377-023-01321-0 (PMC10721804; doi:10.1038/s41377-023-01321-0)
Supplement: Supplementary file 1 — Supplementary Information [file 41377_2023_1321_MOESM1_ESM.pdf]

Supplementary Information for

Quantitatively mapping local quality of super-resolution microscopy by rolling Fourier ring correlation

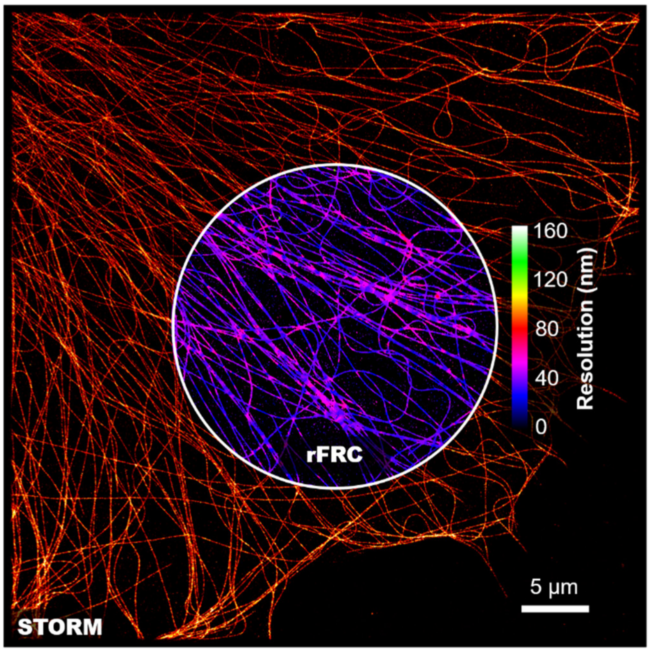

Weisong Zhao, Xiaoshuai Huang, Jianyu Yang, Liying Qu, Guohua Qiu, Yue Zhao, Xinwei Wang, Deer Su, Xumin Ding, Heng Mao, Yaming Jiu, Ying Hu, Jiubin Tan, Shiqun Zhao, Leiting Pan, Liangyi Chen & Haoyu Li

Content

Supplementary Figures. ---------- 3
Supplementary Notes. ---------- 15
Supplementary Note 1 | Uncertainty measurement of unbiased estimation. ---------- 15
Supplementary Note 2 | The stability and resolvability of rFRC map. ---------- 22
Supplementary Note 3 | Limitations. ---------- 27
References. ---------- 29

Supplementary Figures.

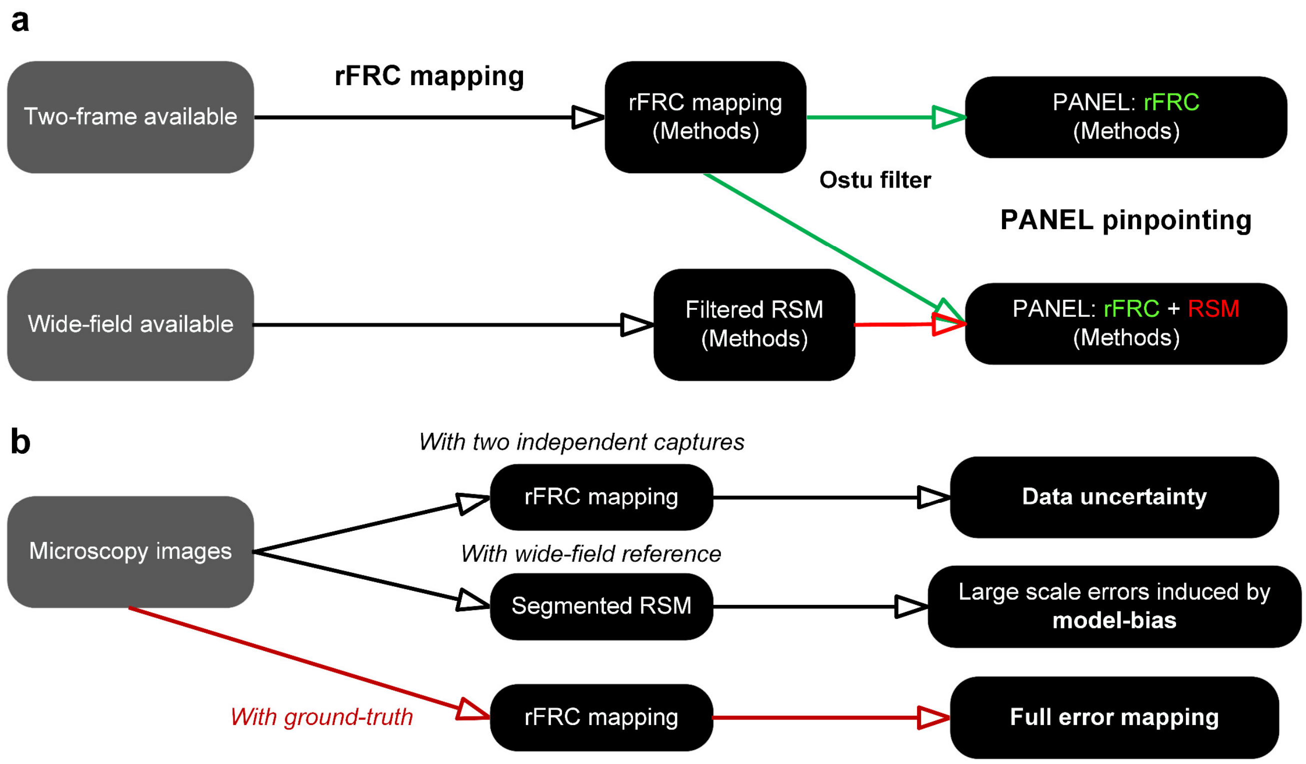

Fig. S1 | Abstract workflow. (**a**) Abstract workflow. Only when the corresponding tasks satisfy two conditions, i.e., (i) belonging to 2D data and (ii) the existence of a wide-field reference, will the RSM be included in the PANEL visualization. (**b**) Our framework for estimating different types of uncertainties. At the SR scale, our method is capable of mapping (i) data uncertainty of image reconstructions without referencing the ground-truth (*Reconstruction-1* vs. *Reconstruction-2*); (ii) large scale errors induced by model-bias referencing wide-field image (*Reconstruction* vs. *Wide-field reference*); and (iii) full error of reconstructions/predictions with ground-truth (*Reconstruction* vs. *Ground-truth*).

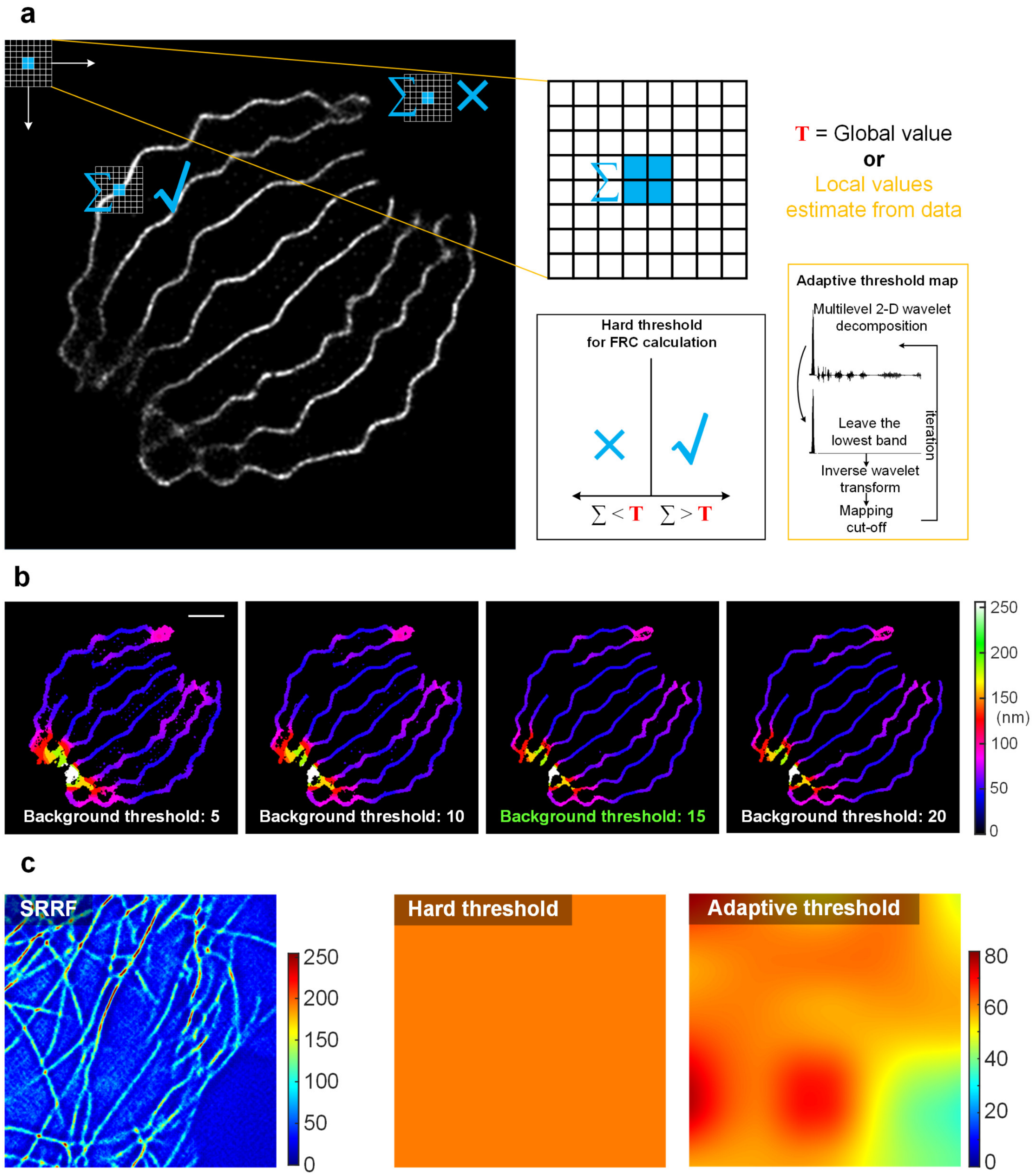

Fig. S2 | Two background skip strategies for rFRC mapping. (**a**) Workflows (*c.f.*, **Fig. 2a**) of the background thresholding methods. During the rolling operation of the rFRC mapping, the intensity of center pixels from each block is summed (blue summation sign). The FRC value is calculated and assigned only if this summed value of the center pixels is larger than (blue tick sign) the threshold ('$\sum > \mathbf{T}$'); otherwise, the center pixel is set to zero (blue cross sign) ('$\sum < \mathbf{T}$'). In this work, we provided two strategies for threshold determination. One is the user-defined hard threshold for the entire image ('15' as in this representative example). The other is the iterative wavelet transform method (yellow box), which automatically estimates the local threshold values. (**b**) rFRC maps using different background thresholds (*c.f.*, **Fig. 2a**). (**c**) A representative SRRF data (left) (*c.f.*, **Fig. S7c**) for illustration of two strategies of background thresholding (middle for hard threshold and right for adaptive threshold).

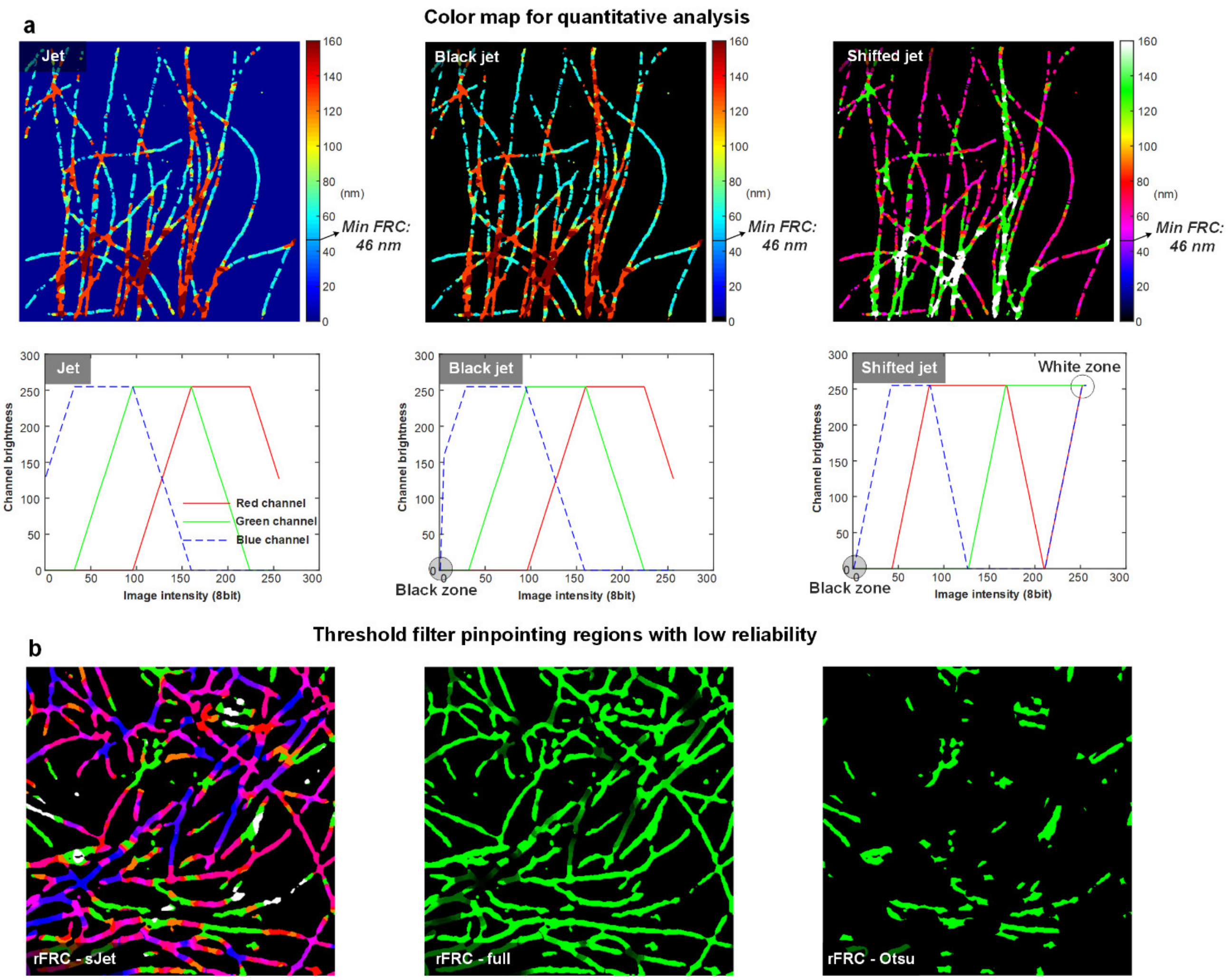

Fig. S3 | Color maps for map display and Otsu threshold for PANEL pinpointing. (**a**) The representative color-coded images and color indexes of jet (left), black jet (middle), and shifted jet (right) color maps. The image is adapted from **Fig. S7a**. (**b**) Otsu threshold for PANEL highlighting. Left: The rFRC map of the SRRF dataset in **Fig. S7c**, displayed in the sJet color map. Middle and right: The Full rFRC map (middle) and the rFRC map after the Otsu threshold (right), regions with low reliability in the SRRF reconstruction are pointed by green.

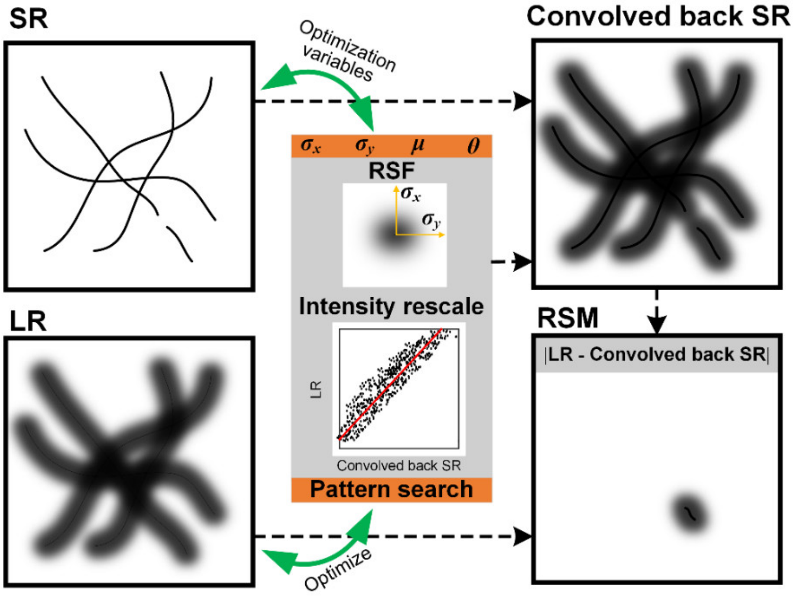

Fig. S4 | The workflow of the RSM.

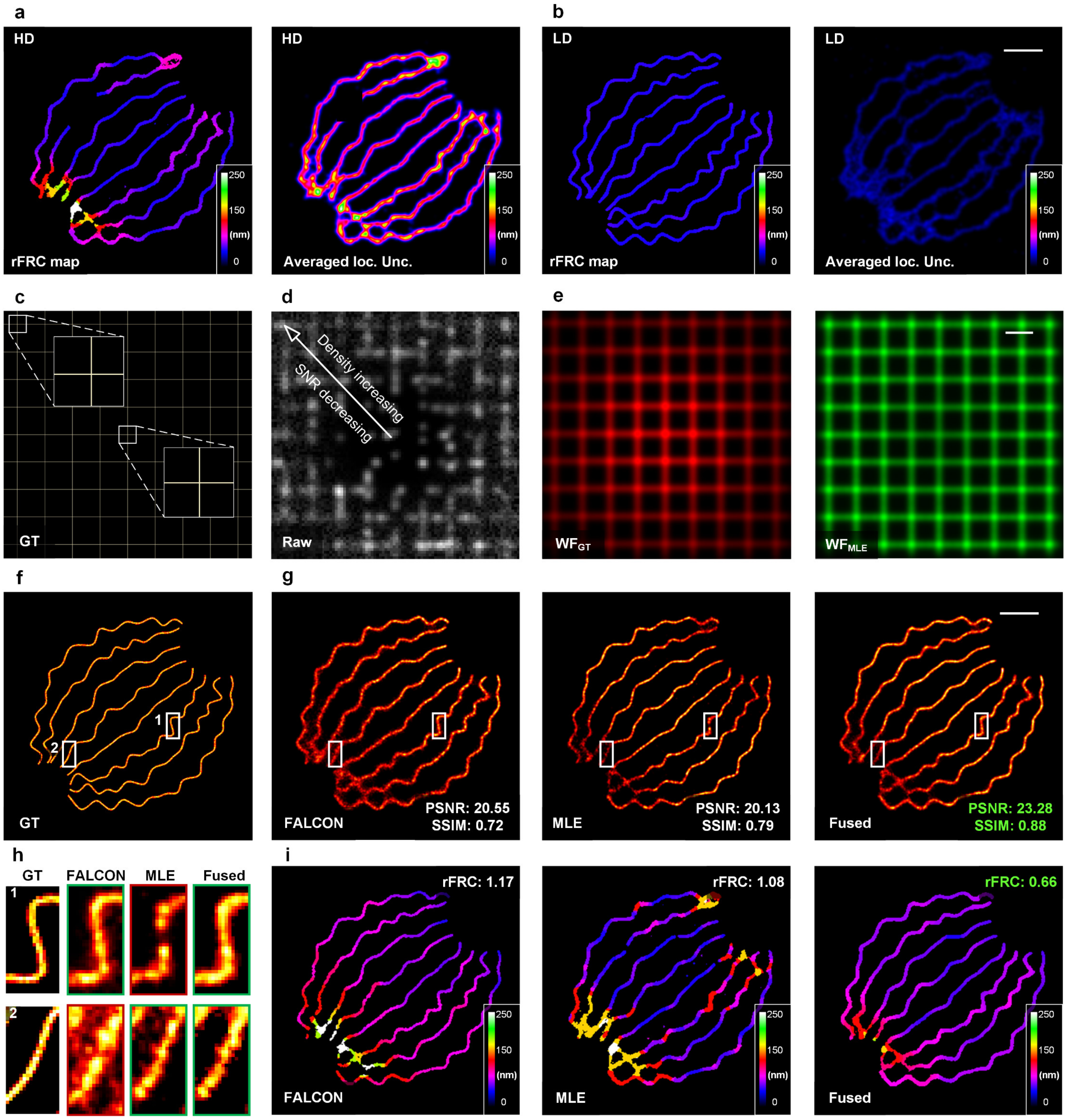

Fig. S5 | Full data of 2D-SMLM simulations and image fusion of SMLM data using rFRC map. (**a**, **b**) The rFRC map versus the localization uncertainty of 2D-SMLM with high-density ('HD', **a**) and low-density ('LD', **b**) emitting fluorophores in each frame (*c.f.*, **Fig. 2a**). The overall resolution distribution of the rFRC map (left) is close to the averaged localization uncertainty map (right). For visualization, we provided the averaged localization uncertainty ('Averaged loc. unc.') map at the right, which is the raw localization uncertainty map filtered with the Gaussian function. (**c**, **d**) The ground-truth structures (**c**), and one representative raw frame (**d**) (*c.f.*, **Fig. 2a**). (**e**) The wide-field ground-truth image (WF_{GT}) and wide-field images generated from the MLE reconstruction (WF_{MLE}). (**f-i**) Image fusion of simulated 2D-SMLM data. (**f**) The ground-truth structures. (**g**) The reconstruction results of FALCON (left) and MLE (middle) algorithms, and the corresponding fused result of these two methods (right). The PSNR and SSIM values (reconstructions versus ground-truth) are labeled on the right bottom. (**h**) Enlarged regions enclosed by white boxes in (**f**) and (**g**). (**i**) rFRC maps of (**g**). Scale bars: (**b**, **g**) 500 nm; (**e**) 1 μm.

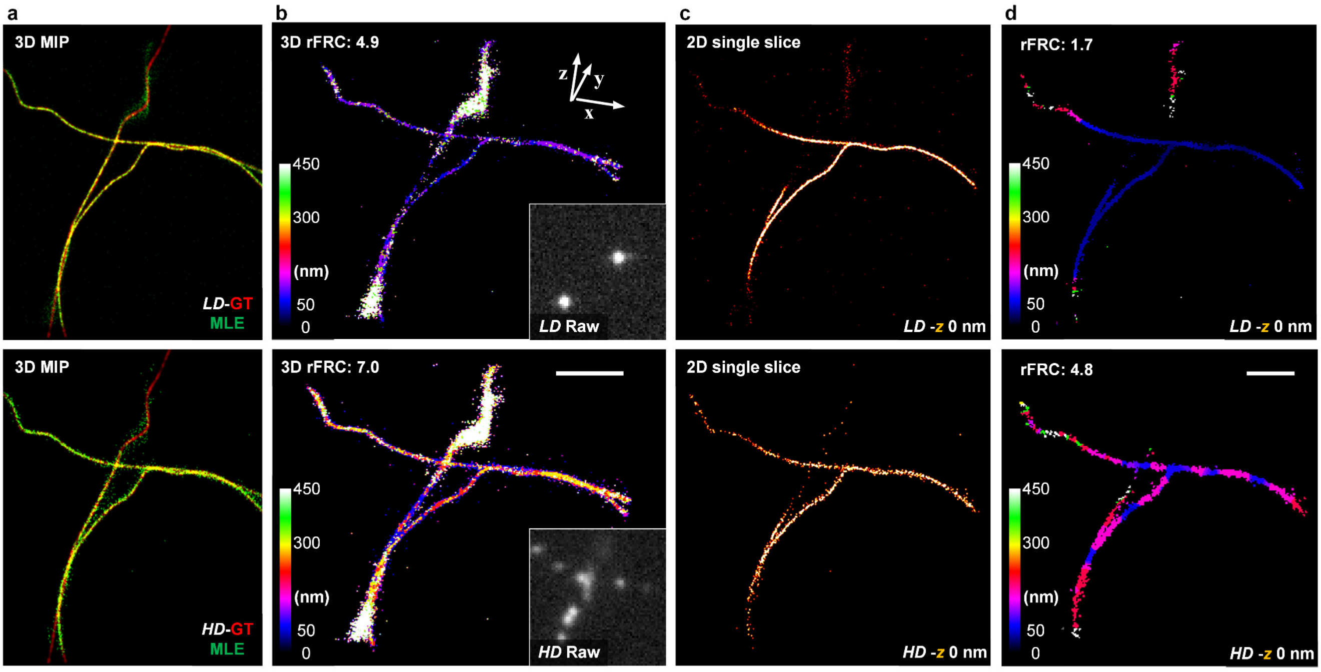

Fig. S6 | 3D-SMLM simulations evaluated by rFRC map. (**a**) The merged maximum intensity projection (MIP) views of ground-truth structures (red channel, labeled as '*LD*-GT' (top) or '*HD*-GT' (bottom) for low-density or high-density emitting fluorophores), and the corresponding 3D-MLE reconstructions (green channel, labeled 'MLE'). (**b**) The rFRC maps of low-density (top) and high-density (bottom) 3D-MLE reconstructions. Insets show representative frames of low-density (top) and high-density (bottom) datasets. (**c**) Horizontal sections (at 0 nm *z* position) of 3D-MLE reconstructions (low-density at the top and high-density at the bottom). (**d**) rFRC maps of corresponding horizontal sections in (**b**). Scale bars: 1 µm.

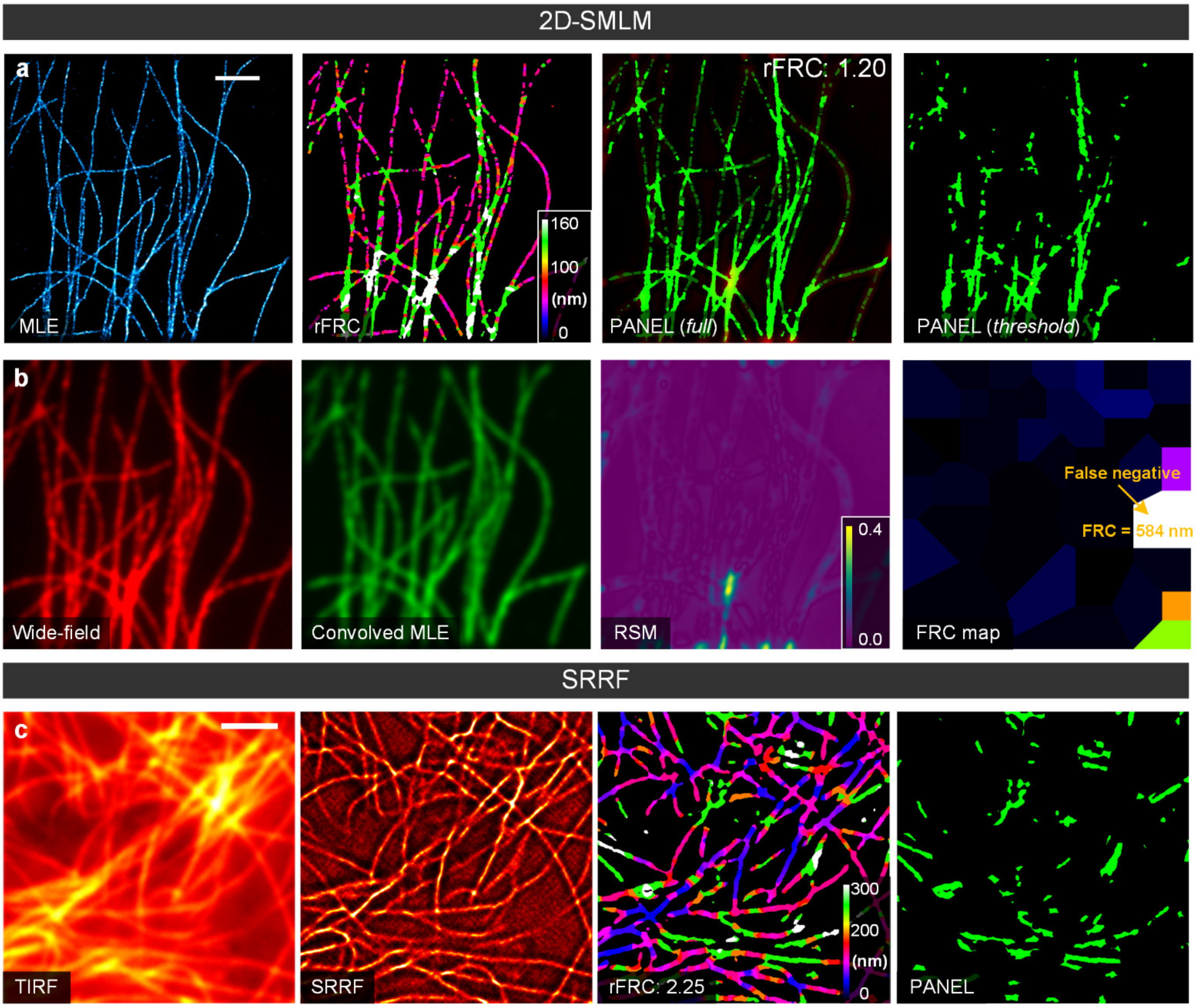

Fig. S7 | Open-source 2D-SMLM and SRRF experimental datasets evaluations. (**a**) From left to right: MLE localization result of 500 high-density images of tubulins from the EPFL website (**Methods**); the rFRC map of the MLE; full merged RSM and rFRC map of the MLE; PANEL visualization. (**b**) From left to right: Corresponding wide-field image; MLE image convolved back to its original low-resolution scale; RSM of the MLE; FRC map of the MLE. (**c**) From left to right: Diffraction-limited TIRF image; SRRF reconstruction result of 100 fluctuation images (GFP-tagged microtubules in live HeLa cells, **Methods**); rFRC map of SRRF; PANEL visualization. Scale bar: 2 μm.

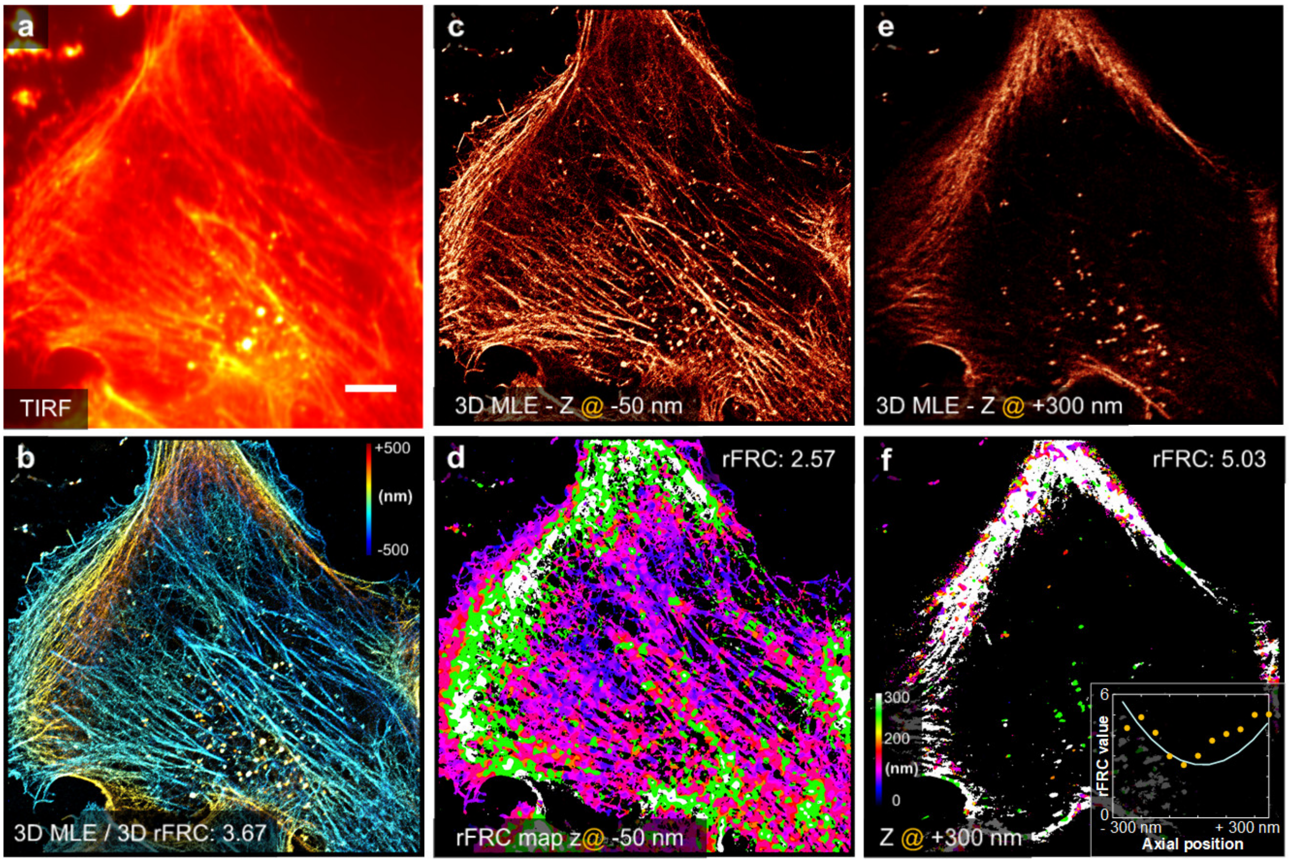

Fig. S8 | Another representative 3D-STORM experiment of phalloidin cytoskeleton evaluated by rFRC map. (**a**) A maximum intensity projection (MIP) view of the TIRF (COS-7 cells, labeled with Alexa Fluor 647-phalloidin). (**b**) Depth color-coded view of 3D-MLE reconstruction. (**c**, **e**) Horizontal section of 3D-MLE reconstruction (**b**) at the -50 nm *z*-position and the corresponding rFRC map (**i**). (**d**, **f**) Horizontal section of 3D-MLE reconstruction (**b**) at the +300 nm *z*-position and the corresponding rFRC map (**k**). Scale bar: (**a**) 5 μm.

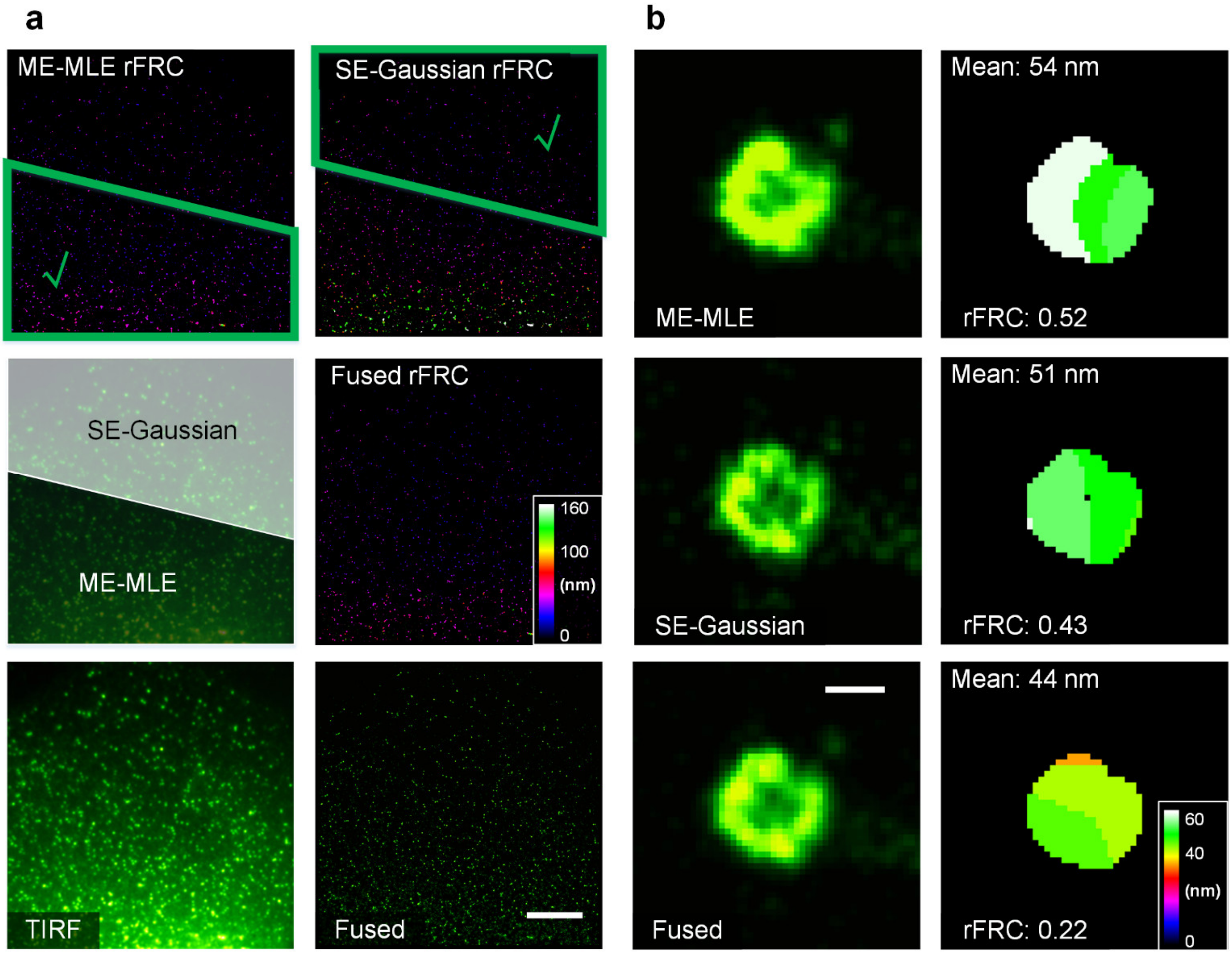

Fig. S9 | Another representative example of STORM fusion (COS-7 cells, heavy chain clathrin-coated pits labeled with Alexa Fluor 647). (**a**) The rFRC map of ME-MLE (top), the superiority map (middle) for fusion, and the TIRF image (bottom) are shown on the left. The rFRC maps of the SE-Gaussian (top) and fusion (middle) results, and the fusion result ('Fused', bottom) are displayed on the right. We found that the ME-MLE method achieves superior performance in the regions containing a strong background and that the SE-Gaussian method obtains better reconstruction quality in the regions containing a weak background. (**b**) Magnified results for a single CCP of ME-MLE (top), SE-Gaussian (middle), and fusion ('Fused,' bottom) are shown on the left, and the corresponding rFRC maps are demonstrated on the right. The mean resolutions are marked on the top left of the rFRC maps. In addition to the stable performance of fusion in the whole field of view, as highlighted in (**a**), the rFRC map assists in fusing fine structures such as a single ring-shaped CCP, enabling higher mean resolution. Scale bars: (**a**) 5 μm; (**b**) 100 nm.

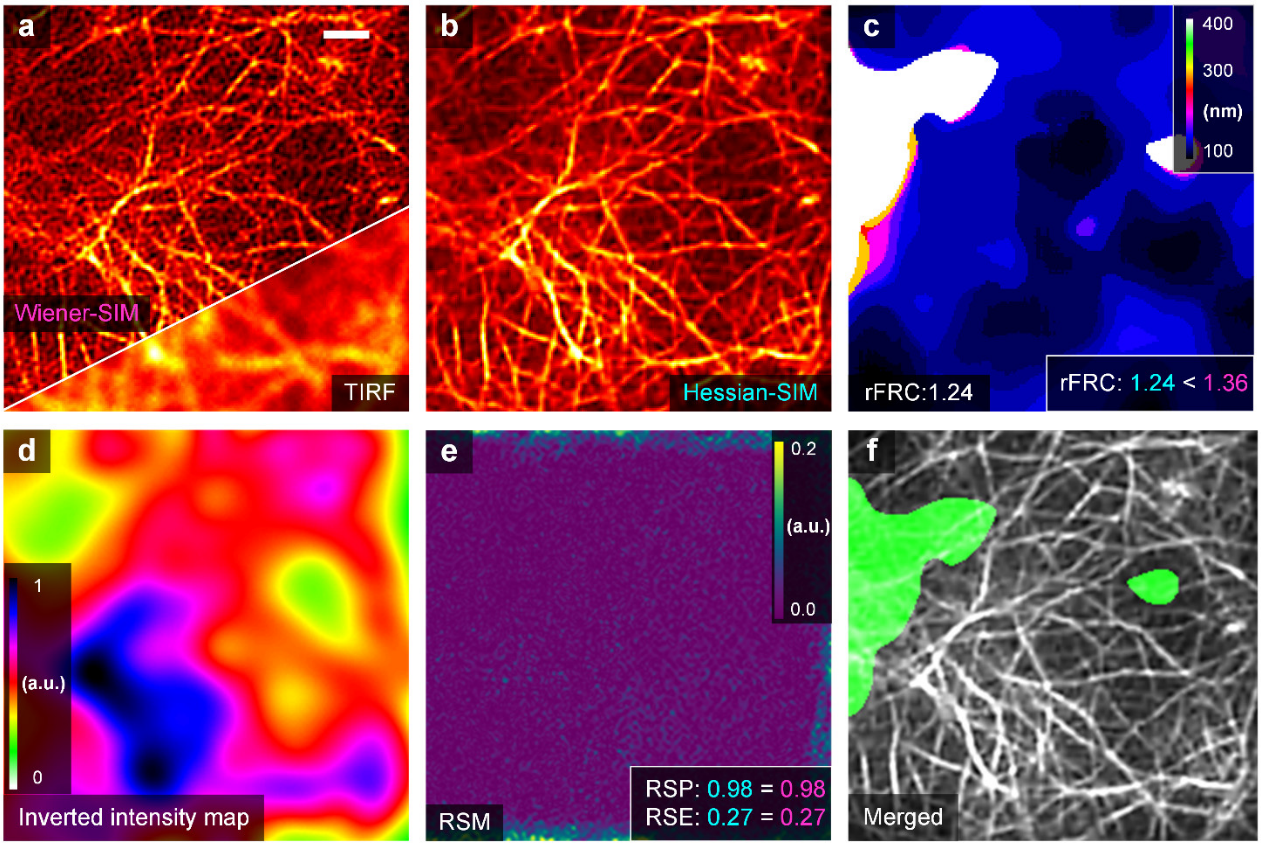

Fig. S10 | Extended visualization of Fig. 6a-6c. (**a**) Representative images of live human umbilical vein endothelial cells (HUVECs) labeled with LifeAct-EGFP under Wiener-SIM (top) and TIRF (bottom) imaging. (**b**) Hessian-SIM result. (**c**) rFRC map of Hessian-SIM. The rFRC, RSP, and RSE values of Wiener-SIM (magenta) and Hessian-SIM (cyan) are shown on the bottom right. Scale bar: 1 µm.

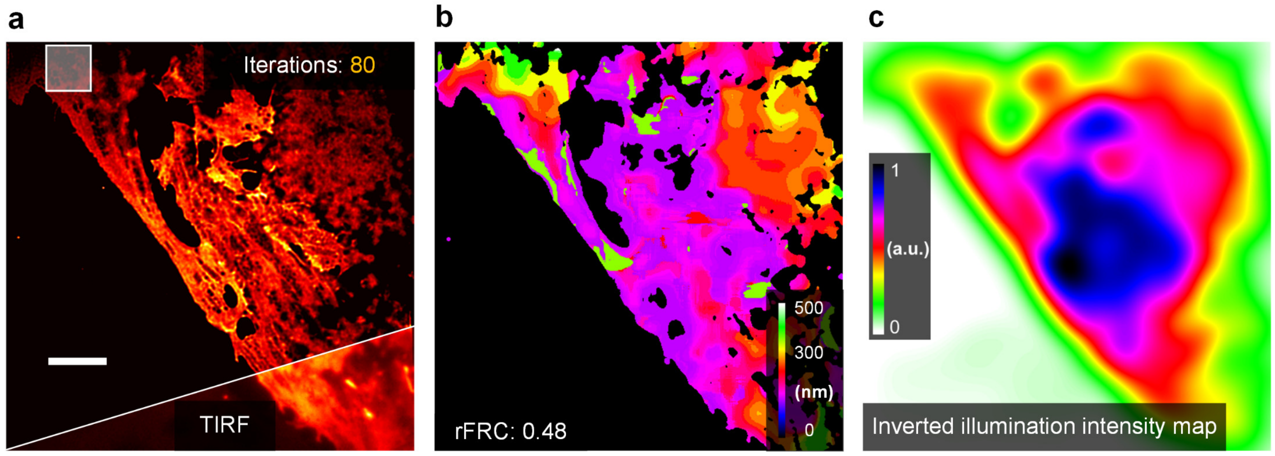

Fig. S11 | Extended visualization of Fig. 6d-6g. (**a**) Representative results of fixed liver sinusoidal endothelial cells (LSECs) labeled with DiI under RL deconvolution (top) and TIRF (bottom) imaging. (**b**) rFRC map of RL deconvolution result. (**c**) TIRF image convoluted with a large Gaussian kernel and coded with an inverted sJet colormap. Scale bar: 5 μm.

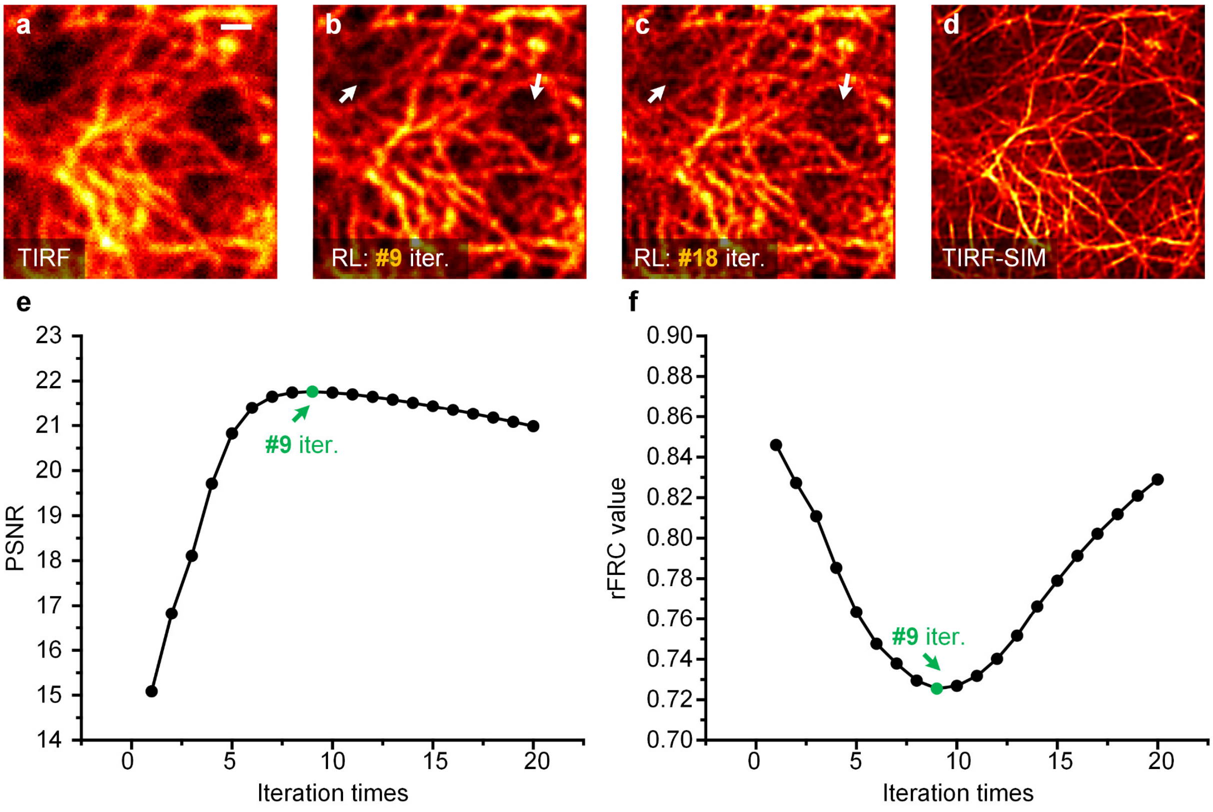

Fig. S12 | Another example of deconvolution iteration determination. (**a-d**) Representative results of live HUVECs labeled with LifeAct-EGFP under TIRF imaging (**a**), RL deconvolution with 9 iterations (**b**), RL deconvolution with 18 iterations (**c**), and TIRF-SIM (**d**). (**e**, **f**) Curves of the PSNR (versus TIRF-SIM) (**e**) and rFRC values (**f**) over iterations. Scale bar: 1 μm.

Supplementary Notes.

Supplementary Note 1 | Uncertainty measurement of unbiased estimation.

Imaging systems use an optical device, mathematically equivalent to transfer functions[1], to observe the objects and then collect the corresponding signals with the sensors. Because the signals are sampled by the sensors[2,3] and corrupted with different types of noise[4-8], the observations always deviate from the real-world objects in the high-dimensional space (left panel of **Fig. S13a**). Thus, recovering the hidden ground-truth signals from the measured data is an ill-posed problem, while each reconstruction represents an approximation deviating from the real-world object to some extent (right panel of **Fig. S13a**). If two measurements are conducted on the same sample and considering the proper reconstruction model used, the distance between the two reconstructions may correlate to the data uncertainty (unbiased estimation, **Fig. S13b**). In this sense, we can use this distance (*Reconstruction 1* versus *Reconstruction 2*) to approximate the magnitude of the reconstructed error.

To test this hypothesis, we restrict the derivation under specific and common circumstances for fluorescence imaging by a wide-field (WF) microscope, where the corresponding image can be processed by the minimalist form of Wiener deconvolution[9].

Lemma. *If we observe the object with an aberration-free, bandwidth-unlimited optical system, in which the object is sampled with an infinite sampling rate without noise, the minimalist form of Wiener deconvolution can recover the object perfectly:*

$$image_{decon} = \tilde{\mathcal{F}}\left\{\frac{H^{*}(\hat{\boldsymbol{\omega}})\cdot\mathcal{F}\left[image_{WF}\right]}{H(\hat{\boldsymbol{\omega}})\cdot H^{*}(\hat{\boldsymbol{\omega}})}\right\} = \tilde{\mathcal{F}}\left\{\frac{\mathcal{F}\left[\left(I(\boldsymbol{\omega})\times o(\boldsymbol{\omega})\right)\otimes h(\boldsymbol{\omega})\right]}{H(\hat{\boldsymbol{\omega}})}\right\} = I(\boldsymbol{\omega})\times o(\boldsymbol{\omega}), \quad (1)$$

where $\boldsymbol{\omega}$ and $\hat{\boldsymbol{\omega}}$ are the spatial and Fourier space coordinates, and *I*, *o*, *h*, and *H* represent the illumination, object, point spreading function (*PSF*), and its Fourier transform of the microscope. $\mathcal{F}$ and $\tilde{\mathcal{F}}$ denote the Fourier and inverse Fourier transform operators, respectively. However, when considering the combined effects of sampling and noise, the model should be expressed as:

$$image_{WF} = \mathcal{N}\left\{\mathcal{S}\left[(I(\boldsymbol{\omega})\times o(\boldsymbol{\omega}))\otimes h(\boldsymbol{\omega})\right]\right\}, \quad (2)$$

where $\mathcal{S}$ and $\mathcal{N}$ denote the sampling and noise model, respectively, and $image_{WF}$ is the final image collected by the WF microscope. This imaging model is considered closer to the status in the real physical world, as shown in **Eq. (2)**, and it is far different from that of the minimalist Wiener model.

$$image_{WF} = \left(I(\boldsymbol{\omega})\times o(\boldsymbol{\omega})\right)\otimes h(\boldsymbol{\omega}). \quad (3)$$

As in **Eq. (1),** we still use the minimalist form of Wiener deconvolution with an aberration-free PSF (unbiased estimation) to process $image_{WF}$, which is given as:

$$
\begin{aligned}
image_{decon} &= \tilde{\mathcal{F}}\left[\frac{H^*(\hat{\boldsymbol{\omega}})\cdot\mathcal{F}\{image_{WF}\}}{H(\hat{\boldsymbol{\omega}})\cdot H^*(\hat{\boldsymbol{\omega}})}\right] \\
&= \tilde{\mathcal{F}}\left|\frac{\mathcal{F}\langle\mathcal{N}\{\mathcal{S}[(I(\boldsymbol{\omega})\times o(\boldsymbol{\omega}))\otimes h(\boldsymbol{\omega})]\}\rangle}{H(\hat{\boldsymbol{\omega}})}\right|. \\
&\neq I(\boldsymbol{\omega})\times o(\boldsymbol{\omega})
\end{aligned}
\tag{4}
$$

The corresponding result $image_{decon}$ in **Eq. (4)** is evidently at some distance from the *Real object*. This **Lemma** implies that the distance between the *Real object* and the reconstruction result is mainly caused by the combined effects of the sampling rates and mixture noise.

Corollary. *Suppose we can control the variables to image the same object and capture a statistically independent image pair. In that case, the difference between the reconstruction and the Real object can be approximated by the distance between reconstructions from the image pair. In other words, this distance (Wiener result$_1$ versus Wiener result$_2$) can be related to the real errors of reconstruction (Wiener result$_1$ and Wiener result$_2$ versus Real object).*

Then, the formula can be given as:

$$\left\|Wiener_1,\ Wiener_2\right\|_{\mathcal{D}} \sim \left\|Wiener_1,\ I(\boldsymbol{\omega})\times o(\boldsymbol{\omega})\right\|_{\mathcal{D}} \cup \left\|Wiener_2,\ I(\boldsymbol{\omega})\times o(\boldsymbol{\omega})\right\|_{\mathcal{D}}, \tag{5}$$

where $\cup$ denotes the union operation (defined as addition or multiplication in this work), and $\left\|Wiener_1, Wiener_2\right\|_{\mathcal{D}}$ represents the distance between the *Wiener result$_1$* and *Wiener result$_2$*. The *Wiener$_n$* is defined as:

$$Wiener_n = \tilde{\mathcal{F}}\left|\frac{\mathcal{F}\langle\mathcal{N}_n\{\mathcal{S}_n[(I(\boldsymbol{\omega})\times o(\boldsymbol{\omega}))\otimes h(\boldsymbol{\omega})]\}\rangle}{H(\hat{\boldsymbol{\omega}})}\right|, \tag{6}$$

where $\mathcal{N}_1$, $\mathcal{N}_2$, $\mathcal{S}_1$ and $\mathcal{S}_2$ are two separate noise models ($\mathcal{N}_1$ and $\mathcal{N}_2$) and sampling models ($\mathcal{S}_1$ and $\mathcal{S}_2$). It is worth noting that the actual model of the union operation from **Eq. (6)** in real-world is complicated, and the combination of the two distances (reconstructions versus real objects) is challenging to express explicitly. For simplicity, we used the **Euclidean distance** or multiplication in the **cross-correlation distance** as approximations.

Euclidean distance. In this example, we use the Euclidean distance to define the distance between two reconstructions and the addition operation to combine the distances. Then, for convenience, we modified **Eq.**

(5) by taking the Fourier transform of the *Wiener results* and *Real object*:

$$\left\|\mathcal{F}\langle Wiener_1\rangle,\ \mathcal{F}\langle Wiener_2\rangle\right\|_2^2 \sim \left\|\mathcal{F}\langle Wiener_1\rangle,\ \mathcal{F}\langle I(\boldsymbol{\omega})\times o(\boldsymbol{\omega})\rangle\right\|_2^2 + \left\|\mathcal{F}\langle Wiener_2\rangle,\ \mathcal{F}\langle I(\boldsymbol{\omega})\times o(\boldsymbol{\omega})\rangle\right\|_2^2, \quad (7)$$

where $\|\cdot,\cdot\|_2$ represents the Euclidean distance. The image is captured with an infinite sampling rate, and it is corrupted with additive noise only, denoted as n_n:

$$\mathcal{F}\langle Wiener_n\rangle = \mathcal{F}\langle I(\boldsymbol{\omega})\times o(\boldsymbol{\omega})\rangle + \frac{\mathcal{F}[n_n]}{H(\hat{\boldsymbol{\omega}})}. \quad (8)$$

By calculating the Euclidean distance, we can simplify **Eq. (7)** to obtain the following form:

$$\sum_{\hat{\boldsymbol{\omega}}}\left\langle\frac{\mathcal{F}[n_1-n_2]}{H(\hat{\boldsymbol{\omega}})}\right\rangle^2 \sim \sum_{\hat{\boldsymbol{\omega}}}\left\langle\frac{\mathcal{F}[n_1]}{H(\hat{\boldsymbol{\omega}})}\right\rangle^2 + \sum_{\hat{\boldsymbol{\omega}}}\left\langle\frac{\mathcal{F}[n_2]}{H(\hat{\boldsymbol{\omega}})}\right\rangle^2, \quad (9)$$

where n_1 and n_2 denote the separate additive noise in the two observations. Then, we simplify **Eq. (9)** as:

$$\sum_{\hat{\boldsymbol{\omega}}}\frac{\mathcal{F}\left[n_1^2+n_2^2\right]}{|H(\hat{\boldsymbol{\omega}})|^2} - \frac{\mathcal{F}[2n_1n_2]}{|H(\hat{\boldsymbol{\omega}})|^2} \sim \sum_{\boldsymbol{\omega}}\frac{\mathcal{F}\left[n_1^2+n_2^2\right]}{|H(\hat{\boldsymbol{\omega}})|^2}. \quad (10)$$

In **Eq. (10)** it clearly shows the close relationship between the distance between the two reconstructions (*Wiener result₁* versus *Wiener result₂*) and the real errors of the two reconstructions (*Wiener result₁* and *Wiener result₂* versus *Real object*).

Cross-correlation distance. Alternatively, if we use the cross-correlation to calculate the distance, the addition operation in the **Euclidean distance** calculation needs to be changed to a multiplication operation:

$$\frac{\sum_{\hat{\boldsymbol{\omega}}}\mathcal{F}_{n_1}\cdot\mathcal{F}_{n_2}^*}{\sqrt{\sum_{\hat{\boldsymbol{\omega}}}\mathcal{F}_{n_1}^2\cdot\sum_{\hat{\boldsymbol{\omega}}}\mathcal{F}_{n_2}^2}} \sim \frac{\sum_{\hat{\boldsymbol{\omega}}}\mathcal{F}_{n_1}\cdot\mathcal{F}_I^*}{\sqrt{\sum_{\hat{\boldsymbol{\omega}}}\mathcal{F}_I^2\cdot\sum_{\hat{\boldsymbol{\omega}}}\mathcal{F}_{n_1}^2}} \times \frac{\sum_{\hat{\boldsymbol{\omega}}}\mathcal{F}_I\cdot\mathcal{F}_{n_2}^*}{\sqrt{\sum_{\hat{\boldsymbol{\omega}}}\mathcal{F}_I^2\cdot\sum_{\hat{\boldsymbol{\omega}}}\mathcal{F}_{n_2}^2}}, \quad (11)$$

where $\mathcal{F}_{n_1}$, $\mathcal{F}_{n_2}$, and $\mathcal{F}_I$ represent the Fourier transforms of *Wiener result₁*, *Wiener result₂*, and the *Real object,* respectively. Then, the following formula can be derived:

$$\frac{\sum_{\hat{\boldsymbol{\omega}}}\mathcal{F}(\mathbf{O}+\mathbf{n}_1)\cdot\mathcal{F}^*(\mathbf{O}+\mathbf{n}_2)}{\sqrt{\sum_{\hat{\boldsymbol{\omega}}}\mathcal{F}(\mathbf{O}+\mathbf{n}_1)^2\cdot\sum_{\hat{\boldsymbol{\omega}}}\mathcal{F}(\mathbf{O}+\mathbf{n}_2)^2}} \sim \frac{\sum_{\hat{\boldsymbol{\omega}}}\mathcal{F}(\mathbf{O}+\mathbf{n}_1)\cdot\mathcal{F}^*(\mathbf{O})\cdot\sum_{\hat{\boldsymbol{\omega}}}\mathcal{F}^*(\mathbf{O}+\mathbf{n}_2)\cdot\mathcal{F}(\mathbf{O})}{\sqrt{\sum_{\hat{\boldsymbol{\omega}}}\mathcal{F}(\mathbf{O}+\mathbf{n}_1)^2\cdot\sum_{\hat{\boldsymbol{\omega}}}\mathcal{F}(\mathbf{O}+\mathbf{n}_2)^2\cdot\sum_{\hat{\boldsymbol{\omega}}}\mathcal{F}(\mathbf{O})^2}}, \quad (12)$$

where $\mathbf{O} \equiv I(\boldsymbol{\omega})\times o(\boldsymbol{\omega})$, and $n_n \equiv \tilde{\mathcal{F}}\langle\mathcal{F}[n_n]/\mathcal{F}[h(\boldsymbol{\omega})]\rangle$ are defined. Similar to the **Euclidean distance, Eq. (12)** shows the close relationship between the **cross-correlation** (*Wiener result₁* versus *Wiener result₂*) and the multiplication of the **cross-correlation** (*Wiener result₁* versus *Real object* and *Wiener result₂* versus *Real object*).

As shown above, both the **Euclidean distance** and **cross-correlation** can be used to estimate the distance between real objects and the corresponding reconstructions. However, it is not easy to choose a superior one to quantitatively map these distances in multiple dimensions (2D image or 3D volume). Conventional spatial methods, such as the spatial subtraction in the RSM[10], calculate the 'absolute differences'[11]. These methods are prone to false negatives in the distance map upon intensity fluctuations and sample movements[11].

Therefore, we introduced a method to measure the distance between two signals in the Fourier domain, namely, Fourier ring correlation (FRC, **Methods**)[12-14] or spectral signal-to-noise ratio (SSNR), describing the highest acceptable frequency component between two signals. The FRC metric has been used as a practical resolution criterion for super-resolution (SR) fluorescence and electron microscopy. Here, we used it to quantify the similarity (distance) between two signals for its insensitivity to intensity changes and micromovements, and also for its quantifying the 'relative error' or 'saliency-based error' (the highest credible frequency component). Because the aberrations in the system may not change during these two independent observations, these aberration-induced errors (biased estimation, **Fig. S13b**) are difficult to estimate when using simple spatial or frequency methods. Instead, these errors may be visible by the FRC since it defines the most reliable frequency component. Overall, the FRC can be a superior choice to quantify the distance between two signals. Furthermore, in this work, considering the FRC as a global similarity estimation between two images, we extended the FRC metric to a rolling FRC (rFRC, **Methods**) map to provide local distance measurements at the pixel level. Therefore, we can quantitatively map the errors in the multidimensional reconstructed signals without the ground truth.

We compared our developed Fourier domain method, rFRC mapping, with a spatial domain method, standard deviation (STD), for data uncertainty measurement in the application of Richardson-Lucy (RL) deconvolution (**Fig. S14**). We repetitively added independent noise 8 times and then applied RL deconvolution on the resulting 8 raw wide-field images. We first calculated the STD on the 2 deconvolved images. Because of severe intensity fluctuations, it is found that the STD with 2 images failed to highlight the three different noise levels. As a result, for this spatial domain method, at least 8 images for STD calculation are required to assess the data uncertainty without amplifying false negatives. By contrast, the rFRC maps using 2 and 8 images exhibit almost identical distributions and both successfully capture the three levels of reconstruction quality. This test suggests that our rFRC mapping method is more stable and efficient compared to the commonly used spatial domain method.

If the two signals lose the identical component in reconstructions, the rFRC may indicate a false positive. This is a potential pitfall of the rFRC method, which could be compensated for by the modified resolution

scaled error map (RSM, **Methods**) method[10]. Specifically, the RSM is built upon three major assumptions:

(i). Because the RSM requires a diffraction-limited WF image as the reference, it must have a sufficiently high SNR. Otherwise, the noise contained in the WF image may induce false-negative effects.

(ii). The transformation from the SR scale to the low-resolution (LR) scale is approximated as a global spatially invariant Gaussian kernel convolution. If the Gaussian-shaped PSF is not spatially invariant, estimation errors will occur.

(iii). The illumination, emission fluorescence intensity, and background are homogeneous. The produced RSM is weighted by the background, illumination intensity, and emission fluorescence intensity of the corresponding label.

If the assumptions mentioned above do not follow, the RSM may introduce false negatives in the estimated error maps. Assumptions **(i)** and **(ii)** may be satisfied under the configurations of single-molecule localization microscopy (SMLM). For example, the WF reference image of SMLM is created by averaging thousands of blinking frames, which removes the noise and generates a high SNR reference. In addition, because the routine imaging field has a small field of view (FOV), it can be regarded as uniform illumination.

The RSM can only find errors on an LR scale (usually error components of large magnitudes), such as misrepresentation or disappearance of structures. In other words, errors in the SR scale (small-magnitude error components) highlighted by the RSM may not be correct. Therefore, we segment the normalized RSM (value less than 0.5 is set to 0) to remove the corresponding components of small magnitude (**Methods**), leaving possible errors of large magnitude (such as the complete absence of structure) to complement the rFRC map. Finally, we merged the developed rFRC map and the segmented normalized RSM in the green and red channels to generate a complete PANEL visualization (**Methods**).

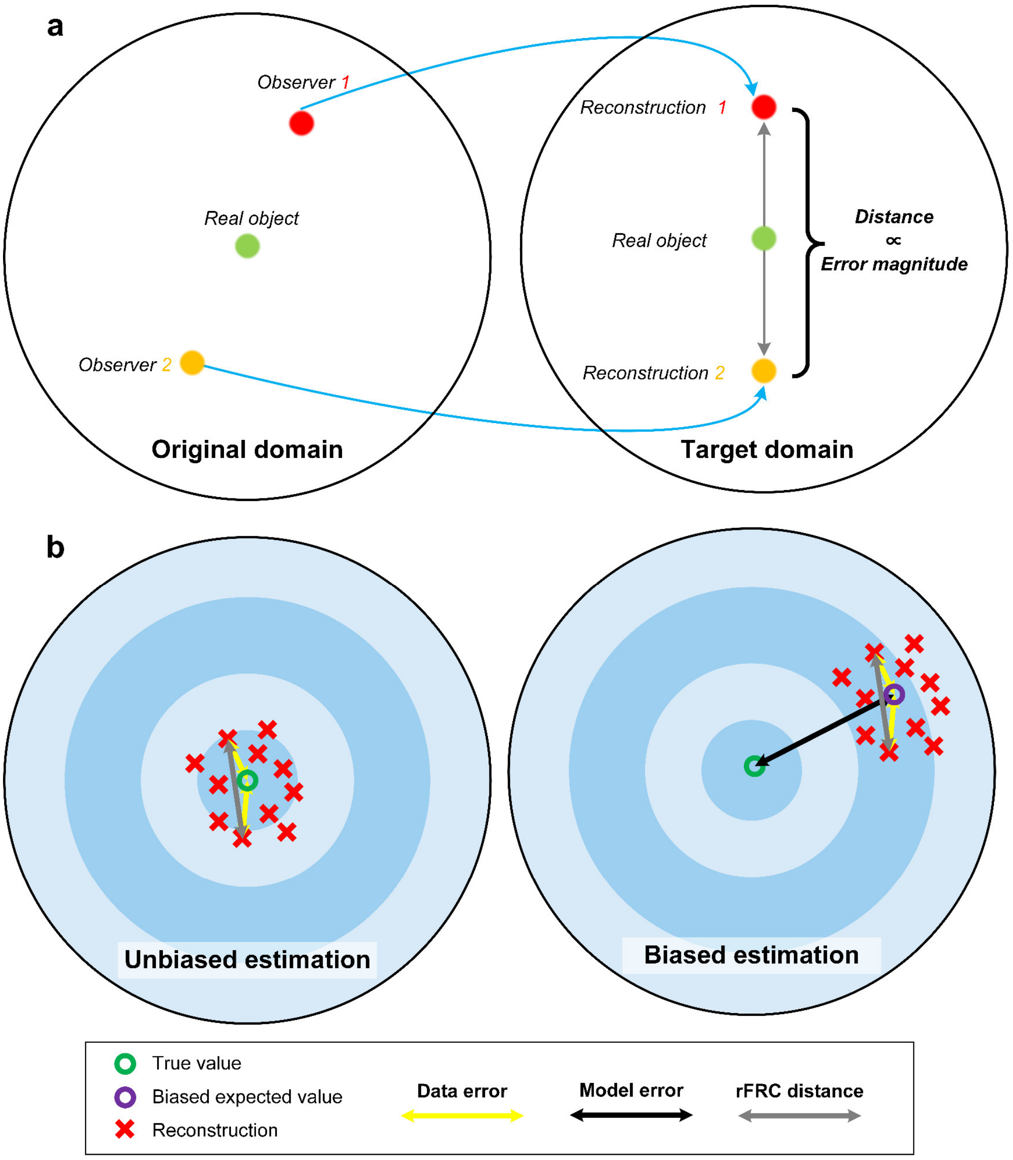

Fig. S13 | rFRC distance. (**a**) The basic concept of rFRC evaluation. The observer in the original domain indicates the captured raw images, and the reconstruction in the target domain represents the reconstructed SR images. (**b**) The data error, the model error, and the rFRC distance. The unbiased estimation indicates that the reconstruction model accurately describes the corresponding real-world model. Then the expected value of reconstructions will be identical to the actual value (green circle). The biased estimation represents that the reconstruction model is different from the real-world one. The expected value (purple circle) of reconstructions will deviate from the actual value (black arrow, model error).

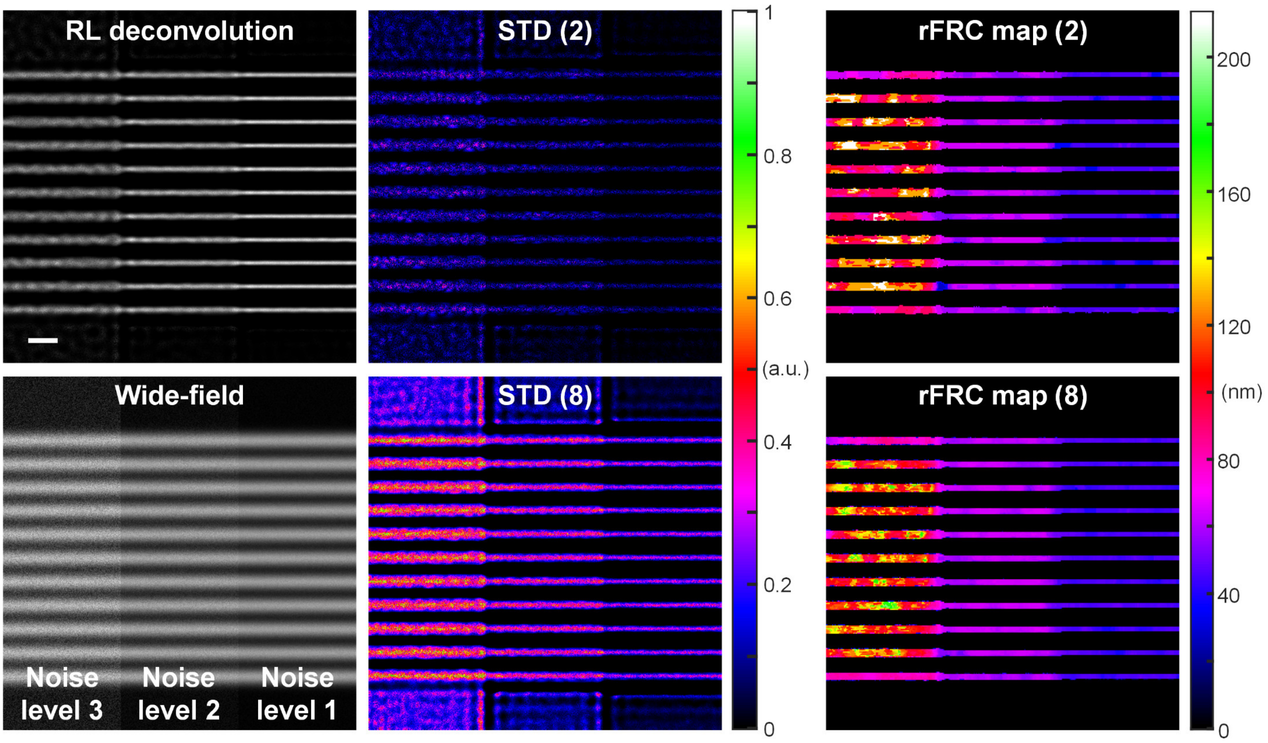

Fig. S14 | Evaluations in spatial domain versus Fourier domain. A series of filaments were convoluted with a wide-field PSF (numerical aperture as 1.4). We gradually decreased the noise level from left to right in three levels (left bottom). Left top: RL deconvolution result. Middle: STD (standard deviation) results of 2 (top) and 8 (bottom) RL deconvolved images. Middle: rFRC map of 2 (top) and 8 (bottom) RL deconvolved images. The 8 images were splitted as 4 batches for rFRC mapping, and the 4 resulting rFRC maps were averaged to create the final rFRC map (8). Scale bar: 500 nm.

Supplementary Note 2 | The stability and resolvability of rFRC map.

Supplementary Note 2.1 | The stability.

To avoid overconfident and unstable determination of the resolutions from small image blocks, we used the 3σ curve[12] as criterion in this work, i.e., three standard deviations above the expected random noise fluctuations as the threshold, instead of the popular-used 1/7 hard threshold[10, 13-15]. This 3σ curve will adaptively change according to the input image block size, and thus it is more stable for local resolution estimation (**Fig. S15a**). To test that, we created images with different block sizes (**Fig. S15b**). In 1/7 hard threshold case, we found that the results were unstable at block size smaller than 256-pixel. Similarly, the 3σ curve from the 32-pixel block did not yield a stable resolution. However, with the larger block sizes (64-, 128, and 256-pixel), the 3σ criterion was stable and remained unchanged around the theoretical resolution. Furthermore, although a smaller block size (e.g., 32-pixel) may lead to more refined mapping, the overall distributions of these rFRC resolution maps (using different block sizes) are close to each other (**Fig. S15c**). Therefore, to balance the mapping scale and its estimation stability, we chose a block size of 64-pixel as default in this work.

Supplementary Note 2.2 | The resolvability.

Fundamentally, the measurement sensitivity of rFRC map (resolvability) is different from the imaging resolution (Rayleigh resolution). When the rFRC mapping is executed by a 64 × 64 block size window, the overlapped image content may indeed induce crosstalk, resulting in a relatively blurry distribution compared to the real one. However, this blurry is in the sense of "resolution", and the "sensitivity" will not be changed profoundly. Because we evaluate the reconstruction quality directly in the SR domain, the quality variations under this blurry can still be identified. For instance, if two adjacent regions are with different reconstruction qualities, this blurry will only lead to a smaller difference in values rather than being completely indistinguishable. In an abstract sense, the reason we can evaluate the reconstruction uncertainties down to SR scale is that we make use of the detection sensitivity rather than the imaging resolution.

To test it and try the maximum resolvability, we simulated structures containing pairs of lines with gradually increasing spacing (**Fig. S16**), and added different noise levels to these paired lines. After that, we applied the rFRC mapping on the resulting images and calculated the FRC resolution distributions of pixels on the left (yellow) and right lines (green) (**Fig. S16**). In the 2-pixel case, the crosstalk between two lines is too significant for the rFRC mapping to distinguish the difference, and thus the overall distributions of FRC resolutions (yellow and green curves) are identical for different noise level. In the 4-pixel case, we observed

the distributions of FRC are just separatable. Paired lines became more separable as overlaps decreased in 8-pixel and 16-pixel cases and were distinct in 32-pixel and 64-pixel cases. Images must satisfy the Nyquist sampling criteria to achieve maximal resolution, so their point spread function (PSF) should cover at least 3-pixel. Therefore, the separation of rFRC of paired lines 4-pixel apart means the minimum detectable scale of rFRC map is up to its limit. By involving the rolling operation, we have addressed a major limitation of the previous FRC map[10], which is challenging to correlate the block-wise map to the SR image content (**Fig. S15c**, **S15d**, and **S17**).

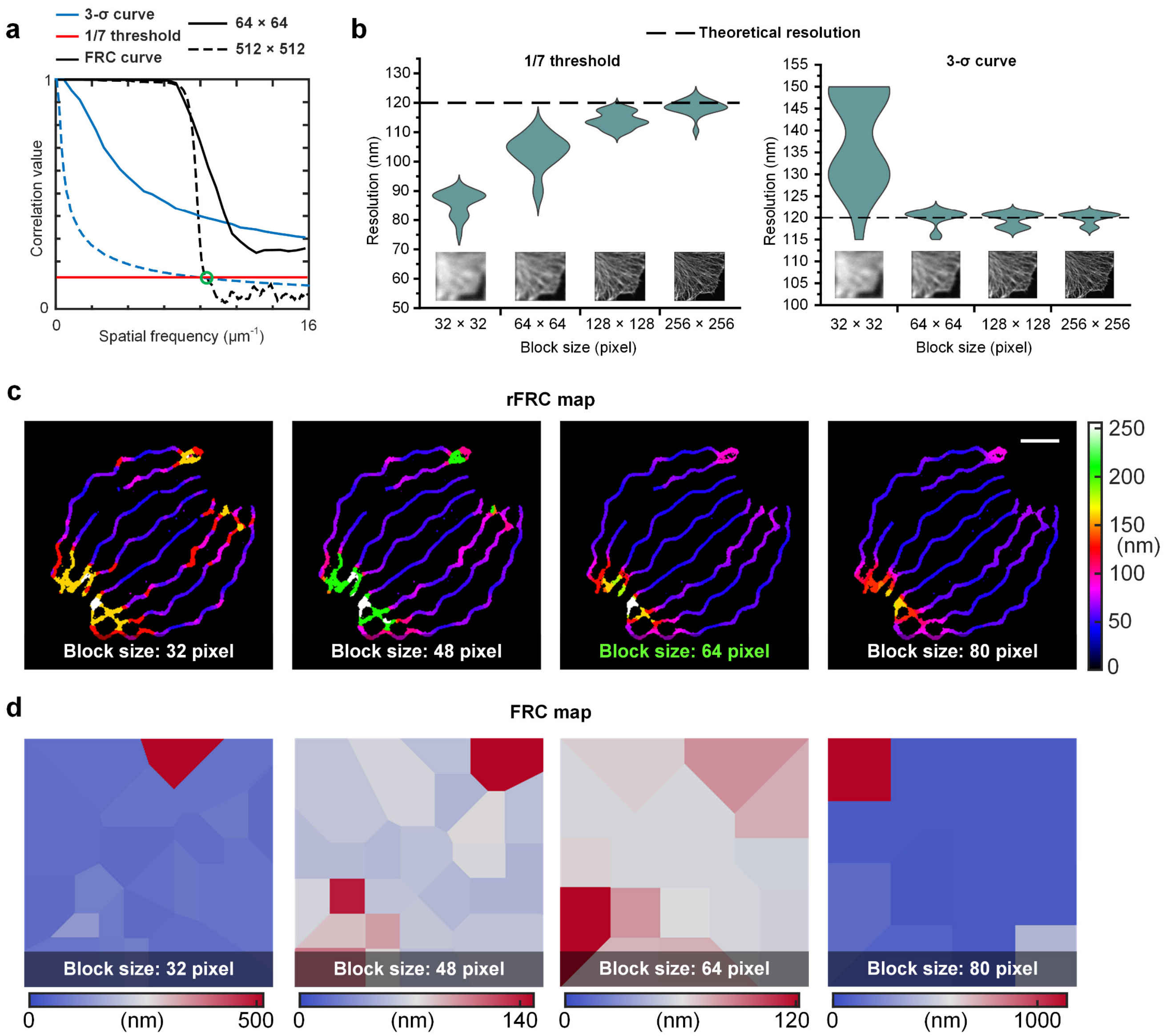

Fig. S15 | The stability of rFRC map. (**a**) The FRC curve (black), 3σ threshold curve (blue), and 1/7 threshold curve (red) for a 64 × 64 pixels image (solid) and a 512 × 512 pixels image (dashed). For an image with a large size (512 × 512 pixels), the 1/7 threshold attains a similar result to the 3σ curve criterion (green circle). However, for a small image (64 × 64 pixels), the 1/7 threshold is smaller than all correlation values in the FRC curve, failing to yield the cutoff frequency. (**b**) The uncertainty of FRC calculation using different block sizes by 1/7 threshold curve (left) and 3σ threshold curve (right). We downsampled the 2D-STORM captured microtubule image (*c.f.*, **Fig. 3f**, 10 nm pixel size, 4096 pixel-number) with 16, 32, 64, and 128 times to create different image sizes and convoluted the resulting images with a 120 nm PSF. After that, Poisson and 5% Gaussian noise were injected into the image. This procedure was repeated 20 times independently and the FRC calculations were performed with different criteria. (**c**) rFRC maps using different block sizes (*c.f.*, **Fig. 2a**). Although the smaller block size (e.g., 32 × 32 pixels) may enable finer mapping, the overall distributions of these rFRC resolution maps using different block sizes are close to each other. On the other hand, the overly small block size may lead to an overconfident resolution value and larger uncertainty. Therefore, to balance the compromise between mapping scale and estimation stability, we chose a block size of 64 × 64 pixels as default in this work. (**d**) FRC maps using different block sizes. Scale bar: 500 nm.

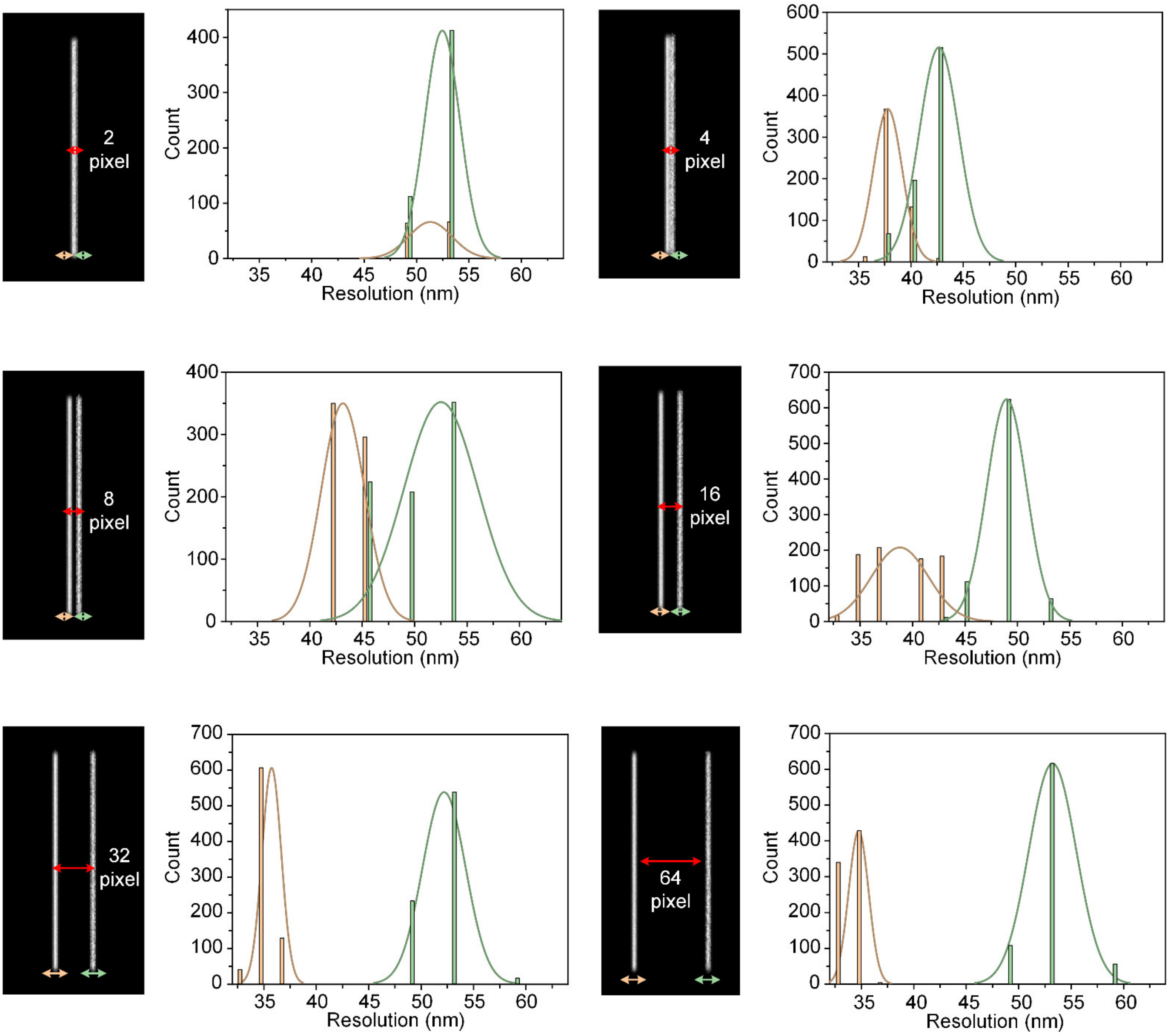

Fig. S16 | The resolvability of rFRC map. We simulated structures that contained pairs of lines with spacing gradually increases, i.e., 2, 4, 8, 16, 32, 64 pixels (pointed by red arrows), and convoluted them by a PSF with a 4-pixel FWHM (pixel size 10 nm). To test the maximum resolvability of rFRC, we included different noise levels on the two lines. Specifically, we added 10% and 50% Gaussian noise on the left and right lines, respectively. After that, we applied rFRC mapping on the resulting images (left panel) and calculated the FRC value distributions (right panel) of pixels on the left (yellow) and right lines (green).

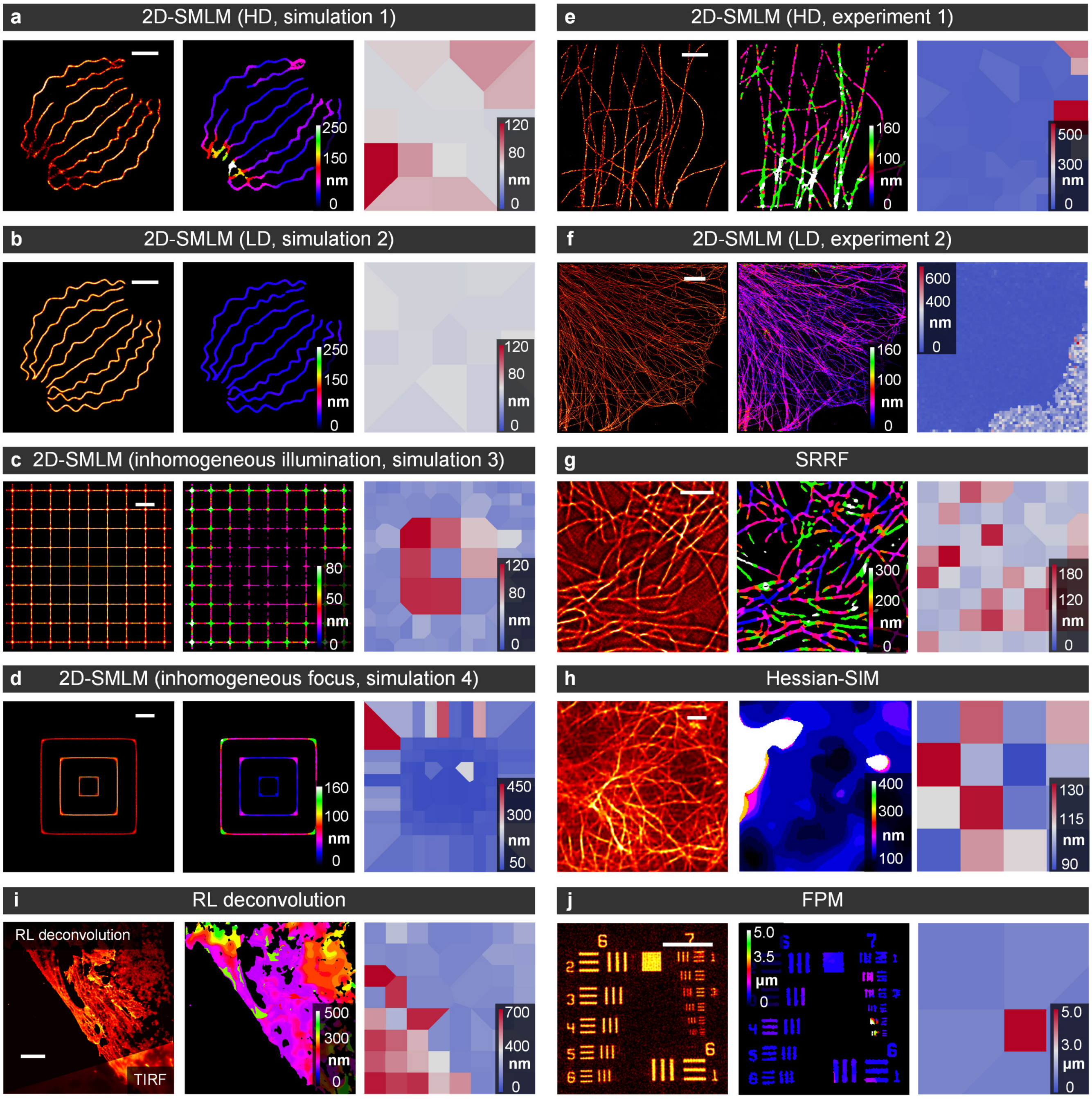

Fig. S17 | rFRC maps versus FRC maps from different modalities. From left to right: Imaging data, rFRC map, and FRC map. (**a**, **b**) 2D-SMLM simulations with high-density ('HD', **a**) and low-density ('LD', **b**) emitting fluorophores in each frame (*c.f.*, **Fig. 2a**). (**c**) 2D-SMLM simulation with inhomogeneous illumination (*c.f.*, **Fig. 2b**). (**d**) 2D-SMLM simulation with inhomogeneous focus (*c.f.*, **Fig. 2c**). (**e**, **f**) 2D-SMLM experiments with high-density ('HD', **e**) (*c.f.*, **Fig. S7a**) and low-density ('LD', **f**) (*c.f.*, **Fig. 3f**) emitting fluorophores in each frame. (**g**) SRRF experiment (*c.f.*, **Fig. S7c**). (**h**) Hessian-SIM experiment (*c.f.*, **Fig. 6a**). (**i**) RL deconvolution experiment (*c.f.*, **Fig. 6d**). (**j**) FPM simulation (*c.f.*, **Fig. 6h**). The FRC map is based on the 1/7 fixed threshold, which may generate unstable calculations. Hence, an inverse distance weight function is involved in interpolating values in all FOVs, while the FRC resolution might not be obtained. This strategy and the calculation on background areas may generate strong false negatives in the resulting FRC map. Scale bars: (**a**, **b**) 500 nm; (**c**, **d**, **h**) 1 µm; (**e**, **g**) 2 µm; (**f**, **i**) 5 µm. (**j**) 50 µm

Supplementary Note 3 | Limitations.

In this part, we discussed the possible caveats of the rFRC and RSM maps, revealing the holistic view of our quantification, including its inherent limitations. Inspired by the Bayesian neural network[16], we defined two major types of uncertainty: the data uncertainty and the model uncertainty. The data uncertainty is mainly induced by the combined effects of noise/sampling. The model uncertainty is primarily caused by the existing distance between the established model and its real-world counterpart. Based on the underlying theory of the corresponding models, the model-related bias can be detected and reduced by careful system calibration[17-19] in optical imaging. On the other hand, data uncertainty is fundamentally model-independent, inevitable, and difficult to remove by system calibration. Our model-independent rFRC can measure the data uncertainty to reflect the data error, and is limited to uncover the model uncertainty-induced reconstruction quality deterioration.

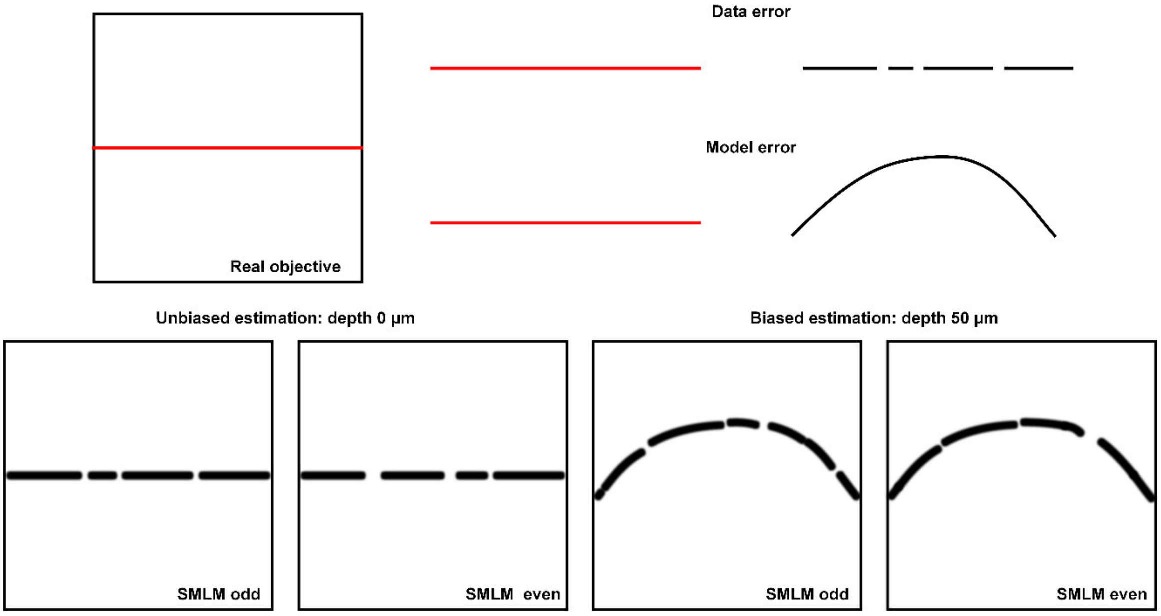

Fig. S18 | Unbiased and biased estimations of SMLM imaging. The straight-line SMLM example enables visualizing unbiased and biased estimations. The real object is a straight line, and the PSF model is approximated as a Gaussian function. At a depth of 0 μm, such PSF model approximation is close enough to the real world (unbiased estimation) model reconstructing the straight-line structure. However, at a depth of 50 μm, due to the index mismatch and sample-induced aberrations, the real-world PSF model will deviate from the point-source Gaussian function. Therefore, the reconstruction (still using the Gaussian function) will distort the straight-line structure to a tilted line (biased estimation). In this case, such model uncertainties cannot be directly detected by the rFRC map.

For example, the PSF mismatch (theoretical PSF versus real PSF) induced by instrument imperfections and the sample-induced aberrations will introduce the biased estimation in SMLM, which can be compensated for by careful system calibration or the *in situ* point spread function retrieval (INSPR)[20]. Using a straight line as an example, if we localize it with the mismatched PSF, the reconstruction will be biased toward structural

distortion. This model uncertainty is visible as seen in **Fig. S18** (structural distortion, the original straight line to a tilted line), which could not be detected by our rFRC.

Supplementary Note 2.1 | Limitations of the rFRC map.

Limitation 1: The normal rFRC method requires two statistically independent images with exact details.

Limitation 2: If the errors are fixed patterns induced by the biased reconstruction model, they will be ignored in the rFRC map. For example, the rFRC using two independent captures cannot reveal errors due to the absence of the identical component in two measurements simultaneously. This limit is complemented by the RSM method to some extent.

Limitation 3: The rFRC can assess local qualities up to (≥) the corresponding SR scale. If the errors are smaller than the SR scale, such as the snowflake-like artifacts, they will be ignored in the rFRC map.

Supplementary Note 2.2 | Limitations of the modified RSM.

Limitation 1: The RSM converts the SR image to its low-resolution scale; thus, it can detect only low-resolution errors. In contrast, errors at the SR scale (small-magnitude error components) estimated by the RSM may be false negatives.

Limitation 2: The RSM map is the absolute residual image between the I_L and I_{HS}. This map is highly corrupted by the intensity and illumination, leading to incorrect quantifications.

Limitation 3: The RSM requires a high-SNR wide-field low-resolution image as a reference.

Limitation 4: The spatially invariant 2D Gaussian kernel convolution assumption may not apply to any optical system, not only introducing false negatives, but also limiting its application to 3D or non-Gaussian convolution data (e.g., denoising applications).

Here, we used a hard threshold of 0.5 in the RSM to detect only significant errors. The removal of minor errors reduces the number of potential false negatives posed by **Limitations 1**, **2**, and **4**. In addition, we apply the complemented rFRC map, which compensates for **Limitation 1** of RSM.

References.

1. Born, M. & Wolf, E. Principles of optics, Edn. 7th. (Cambridge University Press, Cambridge; 1999).
2. Heintzmann, R. & Sheppard, C. The sampling limit in fluorescence microscopy. *Micron* **38**, 145-149 (2007).
3. Lohmann, A. et al. Space bandwidth product of optical signals and systems. *Journal of The Optical Society of America A* **13**, 470-473 (1996).
4. Aspelmeier, T., Egner, A. & Munk, A. Modern Statistical Challenges in High-Resolution Fluorescence Microscopy. *Annual Review of Statistics and Its Application* **2**, 163–202 (2015).
5. Foi, A., Trimeche, M., Katkovnik, V. & Egiazarian, K. Practical Poissonian-Gaussian Noise Modeling and Fitting for Single-Image Raw-Data. *IEEE Transactions on Image Processing* **17**, 1737-1754 (2008).
6. Robbins, M. & Hadwen, B.J. The noise performance of electron multiplying charge-coupled devices. *IEEE Transactions on Electron Devices* **50**, 1227-1232 (2003).
7. Hirsch, M., Wareham, R.J., Martin-Fernandez, M., Hobson, M.P. & Rolfe, D. A Stochastic Model for Electron Multiplication Charge-Coupled Devices: From Theory to Practice. *PLoS ONE* **8**, e53671 (2013).
8. Liu, S. et al. sCMOS noise-correction algorithm for microscopy images. *Nature Methods* **14**, 760-761 (2017).
9. Brown, R. & Hwang, P. Introduction to random signals and applied kalman filtering, Edn. 3rd. (Wiley, New York; 1997).
10. Culley, S. et al. Quantitative mapping and minimization of super-resolution optical imaging artifacts. *Nature Methods* **15**, 263-266 (2018).
11. Zhai, G. & Min, X. Perceptual image quality assessment: a survey. *Science China Information Sciences* **63**, 1-52 (2020).
12. Heel, M.v. & Schatz, M. Fourier shell correlation threshold criteria. *Journal of Structural Biology* **151**, 250-262 (2005).
13. Nieuwenhuizen, R.P. et al. Measuring image resolution in optical nanoscopy. *Nature Methods* **10**, 557-562 (2013).
14. Tortarolo, G., Castello, M., Diaspro, A., Koho, S. & Vicidomini, G. Evaluating image resolution in stimulated emission depletion microscopy. *Optica* **5**, 32-35 (2018).
15. Koho, S. et al. Fourier ring correlation simplifies image restoration in fluorescence microscopy. *Nature Communications* **10**, 1-9 (2019).
16. Kendall, A. & Gal, Y. What Uncertainties Do We Need in Bayesian Deep Learning for Computer Vision? *Advances in Neural Information Processing Systems*, 5580-5590 (2017).
17. Demmerle, J. et al. Strategic and practical guidelines for successful structured illumination microscopy. *Nature Protocols* **12**, 988-1010 (2017).
18. You, S.y., Chao, J., Cohen, E.A., Ward, E. & Ober, R.J. Microscope calibration protocol for single-molecule microscopy. *Optics Express* **29**, 182-207 (2021).
19. Faklaris, O. et al. Quality assessment in light microscopy for routine use through simple tools and robust metrics. *Journal of Cell Biology* **221**, e202107093 (2022).
20. Xu, F. et al. Three-dimensional nanoscopy of whole cells and tissues with in situ point spread function retrieval. *Nature Methods* **17**, 531 - 540 (2020).
